# Supplementary material for: A computational framework for designing micron-scale crisscross DNA megastructures
Source: Nat Commun. 2026 Jul 17;17:8831. doi: 10.1038/s41467-026-75175-6 (PMC13500642; doi:10.1038/s41467-026-75175-6)
Supplement: Supplementary file 1 [file 41467_2026_75175_MOESM1_ESM.pdf]

Supplementary Information for:

"A computational framework for designing micron-scale crisscross DNA megastructures"

Matthew Aquilina*†,[1,2,3], Florian Katzmeier*†,[1,2,3], Minke A.D. Nijenhuis[1,2,3], Siyuan Stella Wang[1,2,3], Corey Becker[1,2,3,4], Yichen Zhao[1,2,3], Su Hyun Seok[1,2,3], Julie Finkel[1,2,3], Huangchen Cui[1,2,3,5,6], Jaewon Lee[7], Seungwoo Lee[2,7,8,9,10], William M. Shih*[1,2,3]

[1]Department of Cancer Biology, Dana-Farber Cancer Institute, Boston, MA 02215, Massachusetts, USA.
[2]Wyss Institute for Biologically Inspired Engineering, Harvard University, Boston, MA 02215, Massachusetts, USA.
[3]Department of Biological Chemistry and Molecular Pharmacology, Harvard Medical School, Boston, MA 02115, Massachusetts, USA.
[4]Harvard College, Harvard University, Cambridge, Massachusetts, USA.
[5]Current Address: 2nd Physics Institute, University of Stuttgart, Stuttgart, Germany.
[6]Current Address: Max Planck Institute for Solid State Research, Stuttgart, Germany.
[7]KU-KIST Graduate School of Converging Science and Technology, Korea University, Seoul, Republic of Korea.
[8]Center for Opto-Electronic Materials and Devices, Post-Silicon Semiconductor Institute, Korea Institute of Science and Technology (KIST), Seoul, Republic of Korea.
[9]Department of Biomicrosystem Technology, Korea University, Seoul, Republic of Korea.
[10]Department of Integrative Energy Engineering, Korea University, Seoul, Republic of Korea.

†These authors contributed equally as first authors.

*Corresponding authors.

E-mails: matthew_aquilina@dfci.harvard.edu, florian_katzmeier@dfci.harvard.edu,

william.shih@wyss.harvard.edu

Contents

1 Supplementary Note 1: Interpretation and Derivation of the Loss Function 3

1.1 Interpretation 3

1.2 Toy Model Formulation 4

1.3 Derivation 5

1.4 Generalization to Multi-layer Structures 7

1.5 Histogram Form 7

1.6 Predictive Limitations 7

1.6.1 Concentration Dependence 8

2 Supplementary Note 2: Alternative Loss Functions 9

3 Supplementary Note 3: Influence of the Handle Library Size on the Loss 11

3.1 Theoretically Predicted Bond Count Distribution 12

4 Supplementary Note 4: Comparison to DNA Sequence Library Generator Seqwalk 14

5 Supplementary Note 5: Comparing the Evolutionary Algorithm with Random Handle Array Selection 16

6 Supplementary Note 6: Assembly kinetics of megastructures with different Losses 17

7 Supplementary Note 7: Optimal parameter search of the evolutionary algorithm 18

8 Supplementary Figures 20

8.1 Wide Field-of-View Images of Figure 4 Megastructure Gallery 20

8.2 Example TEM Images from Figure 5 36

8.3 Example TEM Images from Figure 6/7 (sunflowers) 40

8.4 Example TEM Images from Figure 7 (ribbon) 46

1 Supplementary Note 1: Interpretation and Derivation of the Loss Function

1.1 Interpretation

Here we provide a thermodynamic interpretation of the loss function introduced in Figure 5 in the main text.

$$\text{Loss} = \frac{1}{-\Delta G} \ln\left(\frac{1}{N_{\text{Pairs}}} \sum_{\text{Pairs}} \sum_{\text{Interactions}} e^{-n_{ijk}\Delta G} \right). \tag{S1}$$

Here, ΔG denotes the change in Gibbs free energy for a single plus-handle binding to a minus-handle, and n_{ijk} parameterizes all bond counts of the parasitic interactions between slats. Notably, we changed the index i of the bond count from the main text to its multi-index form ijk. The outer sum runs over all possible pairs ij of plus-handle bearing slats and minus-handle bearing slats, while the inner sum runs over all possible interactions k between a given pair ij. N_{Pairs} denotes the total number of possible pairs.

As mentioned in the main text, the primary purpose of the Loss function is to classify handle assignments of a megastructure based on its slats' parasitic interactions. Contributions from parasitic interactions with higher bond counts are exponentially penalized and are therefore eliminated by the evolutionary algorithm at earlier stages.

We show that, within the framework of a toy model, the Loss function can be interpreted as an effective bond count of the parasitic interactions. In this framework, we approximate all possible interactions between slats by a single effective reaction between all plus-handle bearing slats X and all minus-handle bearing slats Y, forming a single representative complex Z:

$$X + Y \rightleftharpoons Z.$$

Within this approximation, complex formation is characterized by a single effective change in Gibbs free energy ΔG_{eff}. From ΔG_{eff}, we compute an effective bond count of the parasitic interactions by normalizing with the change in Gibbs free energy ΔG for a single plus-handle binding to a minus-handle. The effective parasitic interaction bond count is thus given by

$$n_{\text{eff}} = \text{Loss} = \frac{\Delta G_{\text{eff}}}{\Delta G}.$$

Specifically, a Loss of $n_{\text{eff}} = 2$ means that plus-handle bearing and minus-handle bearing slats interact as if they could form a single complex with the binding energy corresponding to two handles.

1.2 Toy Model Formulation

We first formulate the model for a simple square-shaped crisscross megastructure, as shown in Figure 1 of the main text. In our designs, plus-handle sequences are restricted to the top helix (h5) of each slat, whereas minus-handle sequences are restricted to the bottom helix (h2). Each plus-handle is designed to be fully complementary to one corresponding minus-handle. Our square design therefore consists of a top layer with 32 slats, each carrying 32 minus-handles on the bottom helix, and a bottom layer with another 32 slats, each carrying 32 plus-handles on the top helix (h5). Thus, for the square structure, we have two groups of slats: one bearing plus-handles and one bearing minus-handles. In this toy model we consider only perfectly complementary plus-handle:minus-handle binding interactions (Type 2 in Figure 1B) and neglect all partially complementary interactions (Type 1 in Figure 1B). Consequently, only plus-handle bearing slats can hybridize with minus-handle–bearing slats, whereas plus-handle:plus-handle and minus-handle:minus-handle interactions are excluded.

We can therefore divide the slats into two groups: X_i with $i = 1 \dots 32$ are the plus-handle bearing slats, and Y_j with $j = 1 \dots 32$ are the minus-handle bearing slats. A specific slat X_i and Y_j can form different binding configurations, which we denote as Z_{ijk}, where the index k labels the different possible alignments of these two slats. These configurations are identified by our algorithm by sliding slat Y_j along slat X_i and detecting positions where complementary plus-handles and minus-handles align (see Figure 5A).

For each configuration Z_{ijk}, we assign n_{ijk}, which corresponds to the number of plus-handle:minus-handle bonds formed between the two slats in that specific configuration. We define the change in Gibbs free energy of binding for the configuration Z_{ijk} as

$$\Delta G_{ijk} = \Delta G^0 + n_{ijk} \, \Delta G, \tag{S2}$$

where ΔG^0 is an initiation penalty for complex formation and ΔG is the Gibbs free energy change associated with a single plus-handle binding to its complementary minus-handle.

In our model we work with dimensionless energies and therefore normalize all free energy values by $RT \approx 0.62$ kcal/mol, using $T = 273 + 37 = 310$ K and $R = 1.987 \times 10^{-3}$ kcal mol^{-1}K^{-1}. We assume for simplicity that all handle sequences have the same binding free energy ΔG. The actual Gibbs free energies of hybridization computed by NUPACK 4.0 (`celsius=37`, `[Na`$^+$`]=0.05 M`, `[Mg`$^{2+}$`]=0.025 M`) lie in the range $[-10$ kcal/mol$, -8.45$ kcal/mol$]$, which corresponds to $[-16.1, -13.6]$ in dimensionless units. However, because the handles are tethered to the slats, their effective binding strength is likely reduced compared to the ideal solution case. To keep the model simple while staying in the right physical range, we use a single heuristic value of $\Delta G = -10$, which captures the correct order of magnitude.

With these definitions at hand, we define the equilibrium constant for dimer formation in a specific binding configuration k of a slat pair (X_i, Y_j) as

$$K_{ijk} = \frac{[Z_{ijk}]}{[X_i][Y_j]} = e^{-\Delta G_{ijk}} = e^{-(\Delta G^0 + n_{ijk}\Delta G)}. \tag{S3}$$

Here, $[Z_{ijk}]$ denotes the concentration of the dimeric complex formed in configuration k, and $[X_i]$ and $[Y_j]$ denote the concentrations of the free, unbound slats. The total concentrations of each slat species are conserved. Thus, the total concentrations $[X_i^0]$ and $[Y_j^0]$ of every plus-handle bearing slat i and minus-handle bearing slat j are given by

$$[X_i^0] = [X_i] + \sum_j \sum_k [Z_{ijk}], \tag{S4}$$

$$[Y_j^0] = [Y_j] + \sum_i \sum_k [Z_{ijk}]. \tag{S5}$$

In practice, slats can form multimeric parasitic complexes in which multiple slats bind together in a disordered manner, potentially leading to macroscopic aggregates, as shown in Figure 1 of the main text. Importantly, the formation of any higher-order aggregate is initiated by dimer formation. Thus, dimerization can be regarded as a sentinel event marking the onset of parasitic multimerization.

1.3 Derivation

First we compute the total concentration of dimeric complexes Z and insert Equation S3:

$$[Z] = \sum_{i=1}^{n_h} \sum_{j=1}^{n_{ah}} \sum_k [Z_{ijk}] = \sum_{i=1}^{n_h} \sum_{j=1}^{n_{ah}} \sum_k K_{ijk}\,[X_i][Y_j] = \sum_{i=1}^{n_h} \sum_{j=1}^{n_{ah}} [X_i][Y_j] \sum_k e^{-\Delta G^0 - n_{ijk}\Delta G}. \tag{S6}$$

Here n_h is the total number of plus-handle bearing slats and n_{ah} is the total number of minus-handle bearing slats.

The total concentrations of plus-handle-bearing ($[X^0]$) and minus-handle–bearing slats ($[Y^0]$) are obtained by summing over all species of the respective type, whether free or bound:

$$[X^0] = \sum_{i=1}^{n_h} [X_i^0] = \sum_{i=1}^{n_h} \left([X_i] + \sum_{j=1}^{n_{ah}} \sum_k [Z_{ijk}] \right) = [X] + [Z], \tag{S7}$$

$$[Y^0] = \sum_{j=1}^{n_{ah}} [Y_j^0] = \sum_{j=1}^{n_{ah}} \left([Y_j] + \sum_{i=1}^{n_h} \sum_k [Z_{ijk}] \right) = [Y] + [Z] \tag{S8}$$

Here we used the definition of $[Z]$ and defined the total free slat concentrations $[X] = \sum_{i=1}^{n_h} [X_i]$ and $[Y] = \sum_{j=1}^{n_{ah}} [Y_j]$.

To find an effective description of the model we now apply a mean-field approximation. We assume that all plus-handle bearing slats X_i interact on average in the same way with all minus-handle–bearing slats Y_j.

This implies that the concentrations of free slats within each group are equal. We therefore write

$$[X_i] = \frac{[X]}{n_h} \qquad \text{and} \qquad [Y_j] = \frac{[Y]}{n_{ah}},$$

where $[X]$ and $[Y]$ denote the total concentrations of plus-handle bearing and minus-handle bearing slats, respectively. Here we replace the individual slat concentrations by their average across each group. Note that we implicitly assume that the initial slat concentrations $[X_i^0]$ and $[Y_j^0]$ are identical within each group, which is typically the case in our experiments. Inserting this into Equation S6 yields

$$[Z] = \sum_{i=1}^{n_h}\sum_{j=1}^{n_{ah}} \frac{[X]}{n_h}\frac{[Y]}{n_{ah}} \sum_k e^{-\Delta G^0 - n_{ijk}\Delta G} = \frac{[X][Y]}{n_h n_{ah}} e^{-\Delta G^0} \sum_{i=1}^{n_h}\sum_{j=1}^{n_{ah}}\sum_k e^{-n_{ijk}\Delta G}. \tag{S9}$$

Dividing this equation by $[X][Y]$ yields a definition of the effective equilibrium constant K_{eff}:

$$K_{\text{eff}} = \frac{[Z]}{[X][Y]} = \frac{1}{n_h n_{ah}} e^{-\Delta G^0} \sum_{i=1}^{n_h}\sum_{j=1}^{n_{ah}}\sum_k e^{-n_{ijk}\Delta G}. \tag{S10}$$

We can now define an effective change in Gibbs free energy ΔG_{eff} from the effective equilibrium constant using the standard thermodynamic relation

$$\Delta G_{\text{eff}} = -\ln\left(\frac{1}{n_h n_{ah}} e^{-\Delta G^0} \sum_{i=1}^{n_h}\sum_{j=1}^{n_{ah}}\sum_k e^{-n_{ijk}\Delta G}\right) \tag{S11}$$

$$\Delta G_{\text{eff}} = \Delta G^0 - \ln\left(\frac{1}{n_h n_{ah}} \sum_{i=1}^{n_h}\sum_{j=1}^{n_{ah}}\sum_k e^{-n_{ijk}\Delta G}\right). \tag{S12}$$

Akin to our definition of the change in Gibbs free energy of binding for the configurations Z_{ijk} in Equation S2, we now define

$$\Delta G_{\text{eff}} = \Delta G^0 + n_{\text{eff}}\,\Delta G, \tag{S13}$$

where n_{eff} is the effective interaction bond count of the parasitic interactions. Comparing this to Equation S12 yields the definition of our loss function:

$$n_{\text{eff}} = \frac{1}{-\Delta G}\ln\left(\frac{1}{n_h n_{ah}} \sum_{i=1}^{n_h}\sum_{j=1}^{n_{ah}}\sum_k e^{-\,n_{ijk}\Delta G}\right) = \text{Loss} \tag{S14}$$

where the product $n_h n_{ah}$ is just the number of pairs N_{pair} from Equation S1.

Lastly, we can restate the effective equilibrium constant using the new definitions:

$$K_{\text{eff}} = \frac{[Z]}{[X][Y]} = e^{-(\Delta G^0 + n_{\text{eff}}\Delta G)}. \tag{S15}$$

1.4 Generalization to Multi-layer Structures

The toy model described above applies only to two-layer structures, where one group of slats carries only plus-handles and the other carries only minus-handles. In multilayer crisscross structures, however, a single slat can have plus-handles on one side (for example, on the top helix) and minus-handles on the other (for example, on the bottom helix). Such slats can therefore take part in parasitic interactions with both plus-handle bearing and minus-handle–bearing slats at the same time.

To extend the model, we keep the idea that the first step of parasitic multimerization is the formation of a plus:minus bond. However, instead of describing binding between whole slats, we now describe binding between individual binding sites. In this version, we interpret X_i and Y_j as the concentrations of free plus-handle sites and free minus-handle sites, respectively. The indices i and j label specific sites on specific slats — for example, X_i could be the handle site on slat p.

With this reinterpretation, the derivation above remains unchanged. The only difference is in how the quantities are understood: X and Y are now the total concentrations of free plus-handle and minus-handle sites in the pool, and Z represents the total number of bonds formed between plus-handle and minus-handle sites. Importantly, the model is no longer restricted to physical dimers of whole slats. It now captures individual bonding events between sites.

1.5 Histogram Form

We note an alternative and computationally convenient representation of Equation S1, which is the form implemented in our software package. We define n_ν as the number of interactions in the pool with bond count ν. In this representation, the Loss, or effective parasitic interaction bond count, is given by

$$\text{Loss} = \frac{1}{-\Delta G} \ln\left(\frac{1}{N_{\text{Pairs}}} \sum_{\nu=0}^{\infty} n_\nu \, e^{-\,\nu \Delta G} \right). \tag{S16}$$

1.6 Predictive Limitations

The primary purpose of the Loss function is to serve as an objective for the evolutionary algorithm. It quantifies parasitic interactions by assigning exponentially increasing penalties to higher bond counts, thereby prioritizing the elimination of the strongest off-target interactions during optimization. This behavior is reflected in the bond count distributions shown in Fig. 6**b** of the main text and Supp. Fig. 2.

While the Loss can be interpreted heuristically as an effective bond count, this interpretation has limited predictive power. In particular, the Loss reliably ranks different handle assignments within a given structure: handle assignments with lower Loss generally yield higher assembly success than those with higher

Loss. However, it likely does not directly provide a quantitative prediction of absolute yield across different geometric crisscross designs. For example, two geometrically distinct structures with handle assignments optimized to have the same Loss may still exhibit different assembly yields. As a result, the Loss is best understood as a design metric for improving handle assignments rather than a predictive model of assembly yield. Furthermore:

- The Loss does not account for slat concentrations. Neither global concentration changes nor variations between individual slats are included in the model. Further detail provided in Section 1.6.1 below.
- The Loss has no predictive power with respect to temperature. For example, the heuristically chosen dimensionless free energy $\Delta G = -10$ likely depends on Temperature T through the enthalpy ΔH and entropy ΔS of strand hybridization, as $\Delta G = \frac{\Delta H}{RT} - \frac{\Delta S}{R}$.
- The Loss does not capture the assembly kinetics or pathways of crisscross assembly. A more complete description would require coupling parasitic interactions to a kinetic model of slat incorporation and growth.
- For multilayer structures, the interpretation of the Loss as an effective bond count becomes less direct. In this case, the Loss no longer describes interactions purely between slats but between binding sites, as a single slat can contribute two binding interfaces. This makes predictions across geometrically distinct structures even more difficult.

1.6.1 Concentration Dependence

We can shed some light on concentration dependence within the framework of our toy model (Section 1.2) for the simple Square structure. To recap, we approximate all binding events between plus-handle and minus-handle bearing slats by a single interaction with effective binding constant

$$K_{\text{eff}} = \frac{[Z]}{[X][Y]} = e^{-(\Delta G^0 + n_{\text{eff}} \Delta G)}. \tag{S17}$$

Here, $[X]$ and $[Y]$ denote the concentrations of free plus-handle and minus-handle bearing slats, respectively, and $[Z]$ is the concentration of parasitic complexes. Within this toy model, we can now compute the fraction of slats not participating in parasitic interactions, i.e. slats that remain free and are available for crisscross polymerization. It is therefore expected that a smaller free fraction slows down crisscross assembly.

For simplicity, we consider the symmetric case $[X^0] = [Y^0] = c_0$ and define the fraction of free slats as $f = \frac{[X]}{[X^0]} = \frac{[Y]}{[Y^0]}$. Using the earlier derived conservation relations $[X^0] = [X] + [Z]$ and $[Y^0] = [Y] + [Z]$, we obtain $[Z] = c_0(1 - f)$. Inserting this into the definition of K_{eff} gives

$$K_{\text{eff}} = \frac{1 - f}{f^2 c_0}, \tag{S18}$$

which can be solved as

$$f = \frac{-1 + \sqrt{1 + 4K_{\text{eff}} c_0}}{2K_{\text{eff}} c_0}. \tag{S19}$$

This already indicates that, even within the framework of the toy model, a dependence on the total slat concentration is implied. It also indicates that designs with different numbers of slats can behave differently, since keeping the concentration of each individual slat fixed changes the total slat concentration.

Importantly, this result is obtained under the assumption that all slats within each group are present at the same concentration. If the slats instead separate into two or more groups with different concentrations, the toy model breaks down. The same is true if statistical variations in individual slat concentrations become large. Thus, the toy model can lose descriptive power when the physical system deviates from the assumptions made. Nevertheless, we believe that even in these cases the Loss remains a useful quantity for optimization with our evolutionary algorithm and for quantifying parasitic interactions.

2 Supplementary Note 2: Alternative Loss Functions

Our Loss function (Equation S14) quantifies parasitic interactions by considering only fully complementary (Type-2) plus:minus handle binding. More detailed loss functions could additionally incorporate partial (Type-1) interactions at the sequence level. To be meaningful, such extensions must reproduce the experimentally observed trends in assembly yield at least as well as the standard Loss used in the main text. The different handle assignments for the Square designs shown in Fig. 5 provide a suitable benchmark for evaluating these alternative formulations.

Experimentally, the assembly yield increases monotonically with decreasing standard Loss. We therefore require that any alternative loss function reproduces this ordering across the handle assignments.

We considered the following approaches to compute a more detailed Loss:

- Utilize NUPACK to quantify both the on-target and off-target plus:minus interactions, and then feed these values into Equation S14. Specifically, we replace the term $-n_{ijk}\Delta G$, which represents the number of bonds in configuration k multiplied by a heuristic dimensionless Gibbs free energy. This term is replaced by the sum of the NUPACK-computed Gibbs free energies of all aligned plus:minus handle pairs in that configuration. We considered two versions, including and excluding Type-1 contributions.
- Quantify off-target interactions for a given parasitic binding configuration by counting the number of complementary nucleotides between aligned handle sequences. These counts are summed over all aligned sequences in this configuration. The result is then averaged over all configurations, and finally averaged over all slat pairs.
- Alternatively, quantify off-target interactions by counting nearest-neighbor complementary base pairs instead of individual complementary nucleotides, using the same averaging procedure over configurations and slat pairs.
- Extend the sequence-level strategies above by allowing relative shifts between the two sequences when evaluating interactions. In this case, the sequences are not restricted to perfectly aligned binding domains, but are systematically offset against each other to capture all possible partial overlaps.

- Sequence-level strategies can be incorporated into our Loss function by substituting the term $-n_{ijk}\Delta G$ accordingly. For each binding configuration of two slats, we compute a sequence-level interaction score by summing either all complementary nucleotides or all nearest-neighbor base pairs between the aligned sequences. This sum replaces the exponent in Eq. S14. To retain comparability with the heuristic value $\Delta G = -10$, we rescale the sequence-level scores. For the complementary-nucleotide approach, the total number of matching bases is multiplied by $-10/7$, such that 7 complementary nucleotides contribute equivalently to one fully complementary handle pair. For the nearest-neighbor approach, the corresponding scaling factor is $-10/6$, reflecting the maximum number of nearest-neighbor pairs.

After applying all of these alternative loss functions on the four Square designs of Fig. 5, we obtained the results plotted in Supp. Fig. 1.

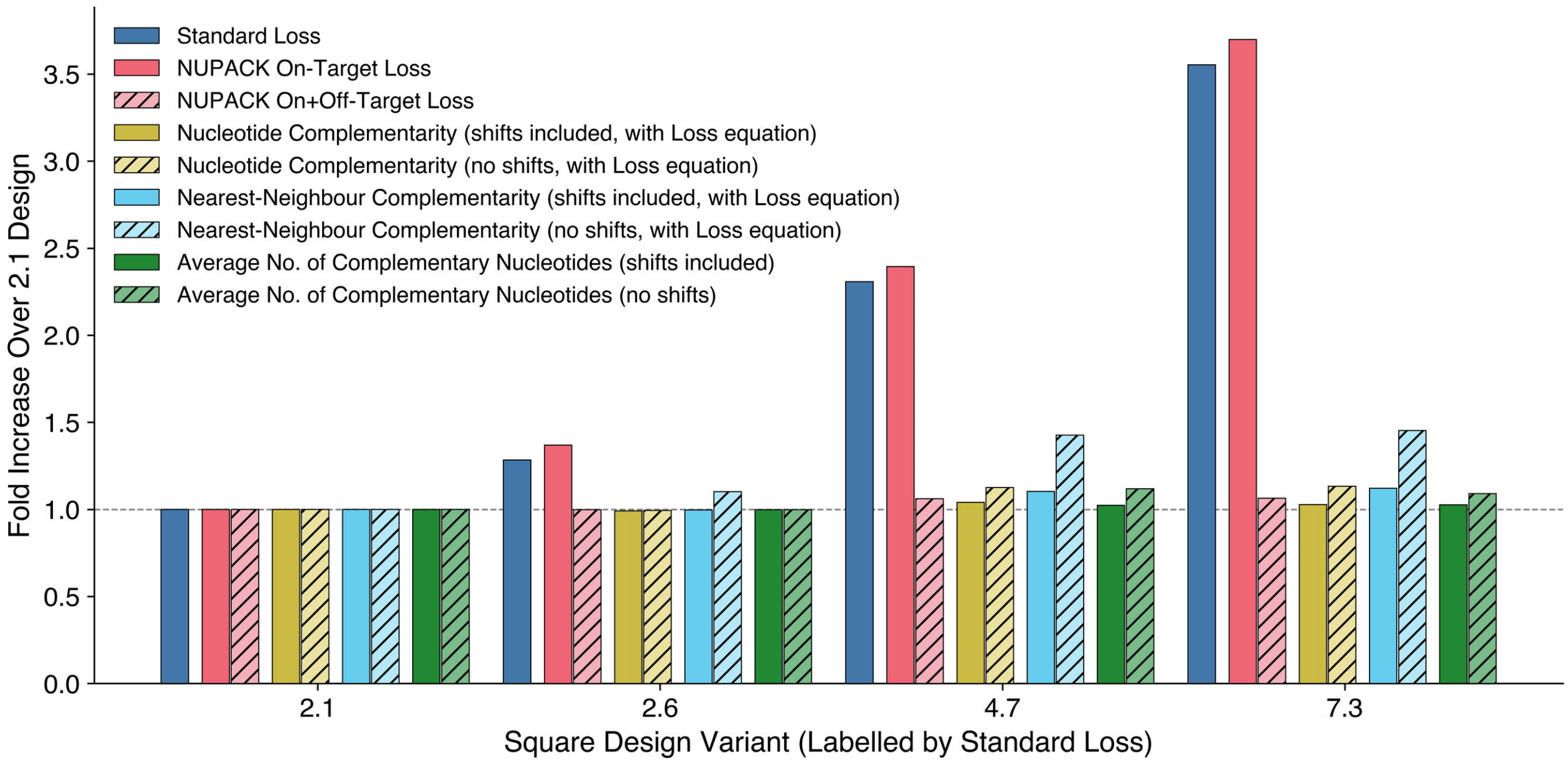

Supp. Fig. 1: Comparison of different methods to quantify parasitic interactions on the Square designs from Fig. 5. The y-axis shows the fold increase in the value of each loss function relative to its value for the structure with the highest experimental yield (standard Loss = 2.1). The four handle assignments are distinguished on the x-axis by our Loss value, labeled as 'Standard Loss' in the legend. Methods that include 'shifts' evaluate interactions over all relative alignments between sequences, i.e., by systematically offsetting one sequence with respect to the other. The number of possible alignments is constrained by the anchoring of handles to the slat backbone. The 'Average No. of Complementary Nucleotides' is a simple average and is not incorporated into the loss equation.

We made the following observations:

- All loss functions assign a lower value to the best-performing handle assignment (standard Loss = 2.1) than to the worst-performing one (standard Loss = 7.4).
- The NUPACK On-Target Loss rates structures similar to our standard Loss as it is simply a more precise measure of on-target Type-2 interactions. Given the very similar distribution, we decided to retain the standard Loss given its significantly reduced compute time and complexity.
- All other loss functions fail to reproduce the ordering predicted by the standard Loss. Specifically, only the standard Loss, the NUPACK on-target Loss, and the nearest-neighbor complementarity Loss without shifts

assign a higher value to the handle assignment with standard Loss = 2.6 than to the design with standard Loss = 2.1.

- The nearest-neighbor complementarity Loss without shifts assigns only a slightly higher value to the design with standard Loss = 7.3 than to the design with standard Loss = 4.7, corresponding to a 1.45- and 1.43-fold increase relative to the handle assignment with standard Loss = 2.1.
- The nearest-neighbor complementarity Loss is the only alternative that accounts for Type-1 interactions and most accurately predicts the experimental yield, but is unable to clearly distinguish between the two worst-performing handle assignments in the series.
- All Loss functions that include shifts were noticeably worse than their non-shift counterparts. This is probably due to them being overly pessimistic on how strong an off-target interaction can be. Similarly, the NUPACK off-target interactions are highly pessimistic and completely smoothed out any differences between handle assignments.

From the results, none of the alternative loss functions we tested could capture the experimentally observed trends, suggesting that Type-2 parasitic bonds are the dominant contributors to parasitic interactions. This is consistent with our observations in Fig. 7 of the main text, where no significant improvement in crisscross assembly speed was found for more orthogonal sequence libraries i.e. with overall reduced Type-1 parasitic binding.

3 Supplementary Note 3: Influence of the Handle Library Size on the Loss

We investigated how the size of the handle library influences the achievable Loss and the corresponding distribution of parasitic interaction bond counts. Two representative designs were analyzed: the Square and the Sunflower.

For each design, we generated handle assignments using libraries containing 8, 16, 32, 48, and 64 handle pairs. For each condition, we evaluated both the initial bond count distribution corresponding to a fully random (unoptimized) handle assignment and the distribution obtained after running 2000 generations of the evolutionary algorithm. The resulting distributions are presented in Supp. Fig. 2.

For both designs, increasing the handle library size leads to a suppression of high bond count parasitic interactions. Specifically, larger handle libraries result in narrower bond count distributions with reduced maximum bond counts, indicating a lower probability of strong parasitic interactions.

The evolutionary algorithm primarily acts by eliminating parasitic interactions with high bond counts, while leaving the overall shape of the distribution largely unchanged. This is best demonstrated using the Square design, where we plotted a black line corresponding to the theoretically predicted distribution of bond counts (explained later). For random assignments, the distributions align with this black line for all conditions. For optimized assignments, the same envelope is preserved, but the highest bond count classes are missing.

Across all tested conditions, the algorithm consistently removes at least the highest two bond count classes from the distribution. Interestingly, for smaller handle libraries (8 and 16 pairs), the improvement achieved by the evolutionary algorithm is more pronounced.

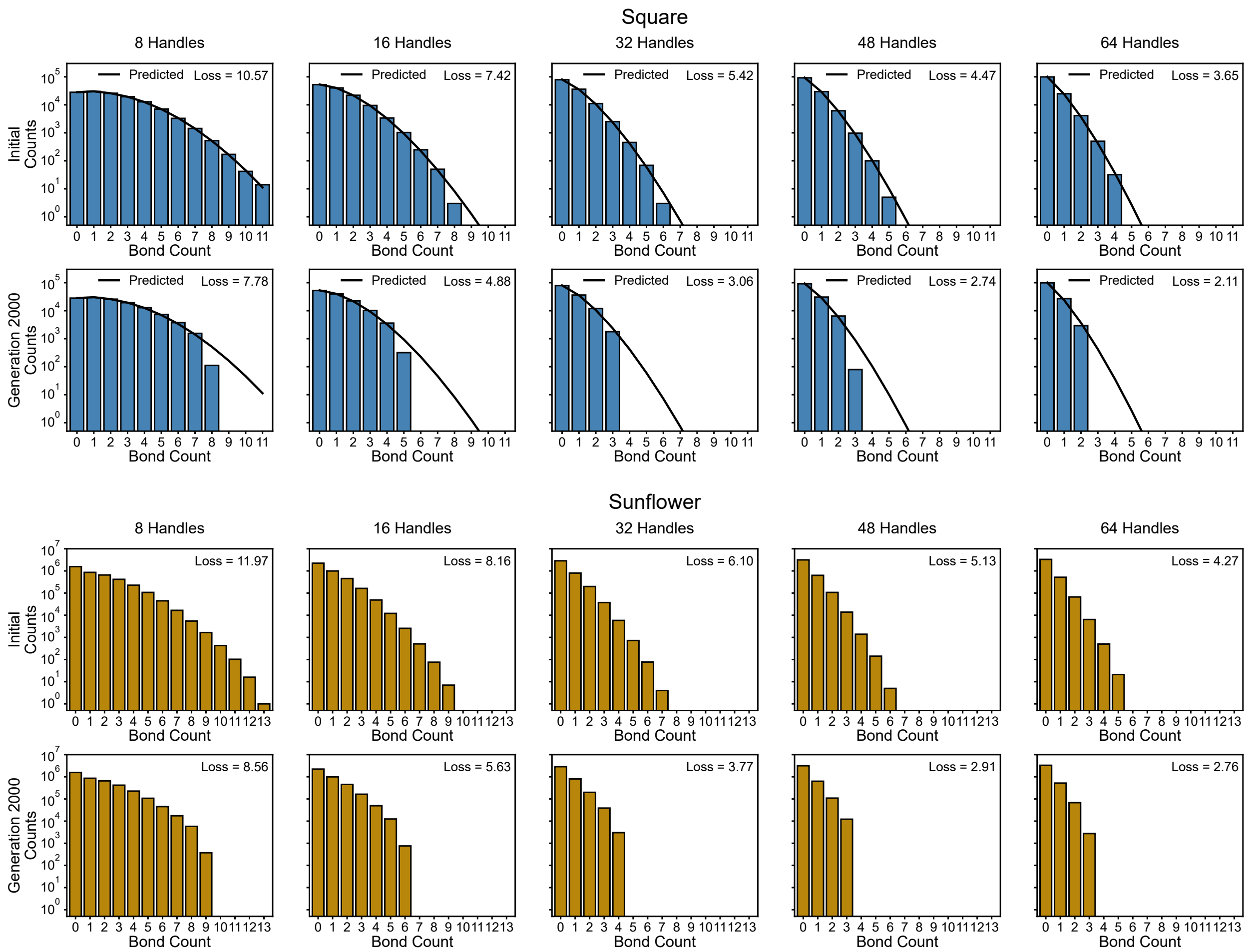

Supp. Fig. 2: Distributions of parasitic interaction bond counts for handle libraries containing 8, 16, 32, 48, and 64 handle pairs. Two designs are shown: the Square (Fig. 5) and Sunflower (Fig. 6). The black line in the Square panels indicates the theoretically predicted distribution for a random handle assignment. All evolutionary optimizations were performed using the same parameter set employed for Fig. 6: 2000 generations, a population size of 50, 3 survivors per generation, a mutation rate of 2 and a p_d of 0.85.

3.1 Theoretically Predicted Bond Count Distribution

For certain two-layer designs such as the Square and the Hexagon, the bond count distribution for fully random handle assignments can be predicted analytically. This is possible because, in these designs, all plus-handle bearing slats and all minus-handle bearing slats have every handle position occupied.

We begin by determining the number of possible bonds $\Omega(k)$ between a plus-handle-bearing slat and a minus-handle-bearing slat for each binding configuration k. The configurations are generated by sliding one slat along the other. The index k denotes the relative shift, where $k = 0$ corresponds to a tip-to-tip configuration with no overlap, and $k = 1$ corresponds to a shift by one position, resulting in a single overlapping handle.

As k increases, the number of overlapping handles increases by one with each shift until the slats fully overlap after l shifts, where l is the slat length. Beyond this point, the overlap decreases symmetrically with further shifts until it returns to zero.

The number of possible bonds is therefore given by:

$$\Omega(k) = \begin{cases} 0, & k < 1 \text{ or } k > 2l-1, \\ k, & 1 \le k \le l, \\ 2l-k, & l < k \le 2l-1, \end{cases} \tag{S20}$$

$$\Omega(k) = \max\bigl(0,\ \min\{\,k,\ l,\ 2l-k\,\}\bigr) \tag{S21}$$

For a given configuration k, the number of formed bonds is binomially distributed with $\Omega(k)$ independent Bernoulli trials, since handles are assigned independently at random. The probability that one handle forms a bond is $p_b = \frac{1}{h}$ for a handle library of size h. Thus, the probability of observing b bonds in configuration k is

$$P(b \mid k) = \binom{\Omega(k)}{b} p_b^{\,b} \,(1-p_b)^{\Omega(k)-b}. \tag{S22}$$

We can now average over all binding configurations k to obtain the probability of observing b bonds in any configuration. Assuming that all relative shifts are equally likely, each configuration occurs with probability $\frac{1}{2l-1}$. Thus, we obtain

$$P(b) = \frac{1}{2l-1} \sum_{k=1}^{2l-1} \binom{\Omega(k)}{b} p_b^{\,b} \,(1-p_b)^{\Omega(k)-b}. \tag{S23}$$

If we have n plus-handle bearing slats and m minus-handle bearing slats, the total number of configurations is given by $2\,n\,m\,(2l-1)$. The factor of two accounts for additional configurations obtained by flipping one slat with respect to the other. Thus, the expected number of configurations resulting in b bonds is given by

$$D(b) = 2\,n\,m\,(2l-1)\,P(b). \tag{S24}$$

Using the expression derived above, we obtain

$$D(b) = 2\,n\,m \sum_{k=1}^{2l-1} \binom{\Omega(k)}{b} p_b^{\,b} \,(1-p_b)^{\Omega(k)-b}. \tag{S25}$$

4 Supplementary Note 4: Comparison to DNA Sequence Library Generator Seqwalk

We investigated whether the recently developed DNA sequence barcode library generator seqwalk also produces orthogonally binding DNA sequences. seqwalk generates DNA sequence pairs such that any substring of length k and its reverse complement appear at most once across all sequences. We tested seqwalk for $k = 3, 4, 5$ and evaluated the resulting sequences using NUPACK. As in the main text (Fig. 7c), we analyzed the distributions of on-target and off-target binding energies.

In Fig. 3, column **a** shows the on-target and off-target energy distributions for the sequences generated by seqwalk. For comparison, column **b** shows the corresponding distributions for randomly generated sequence pairs with the same number of sequences. Column **c** shows the binding energy distributions for the 64 sequences from our sequence library, evaluated both with and without the 5′-TT flanking bases, as seqwalk does not support flanking sequences. Across all plots, the number of identified sequence pairs is indicated in the title. The Gibbs free energy of the worst off-target binding interaction is shown as a vertical dashed line, with its value reported in the legend of each plot.

A clear separation between on-target and off-target energies is observed only for $k = 3$, for which seqwalk identifies just 6 sequence pairs. For $k = 4$ and $k = 5$, larger sets of 23 and 170 sequence pairs are obtained. However, the off-target cutoff lies within the distribution of on-target energies, indicating the absence of a well-defined energy gap. In comparison to random sequence pairs, seqwalk-generated sequences exhibit a consistently lower off-target cutoff. However, the overall energy distributions remain largely similar, with only minor qualitative improvements in binding specificity. In contrast, our optimized 64-sequence library exhibits a pronounced separation between on-target and off-target energies. Moreover, our approach enables control over the on-target energy range, which cannot be enforced using seqwalk.

These results indicate that the application of seqwalk for generating orthogonally binding DNA sequences is limited. This is not unexpected, as seqwalk was not designed with thermodynamic binding optimization as an objective. A specific example illustrates why seqwalk does not reliably produce orthogonally binding sequences. For $k = 4$, the worst off-target interaction occurs between the sequence pairs GCCGACC / GGTCGGC and ACCGCCC / GGGCGGT. In particular, the plus handle GCCGACC binds to the minus handle GGGCGGT with an off-target binding energy of -8.567 kcal/mol. This strong off-target interaction arises from the combination of high GC content and multiple short binding stretches. Specifically, the sequences form two contiguous base-pairing regions of three and two bases, separated by a single mismatch. While each individual k-mer remains unique, these partial overlaps still enable substantial hybridization. Notably, seqwalk enforces global uniqueness of k-mers across the sequence library but does not constrain the number or arrangement of nearest-neighbor interactions in off-target binding. As a result, sequences can satisfy the seqwalk design criteria while still exhibiting significant unintended hybridization.

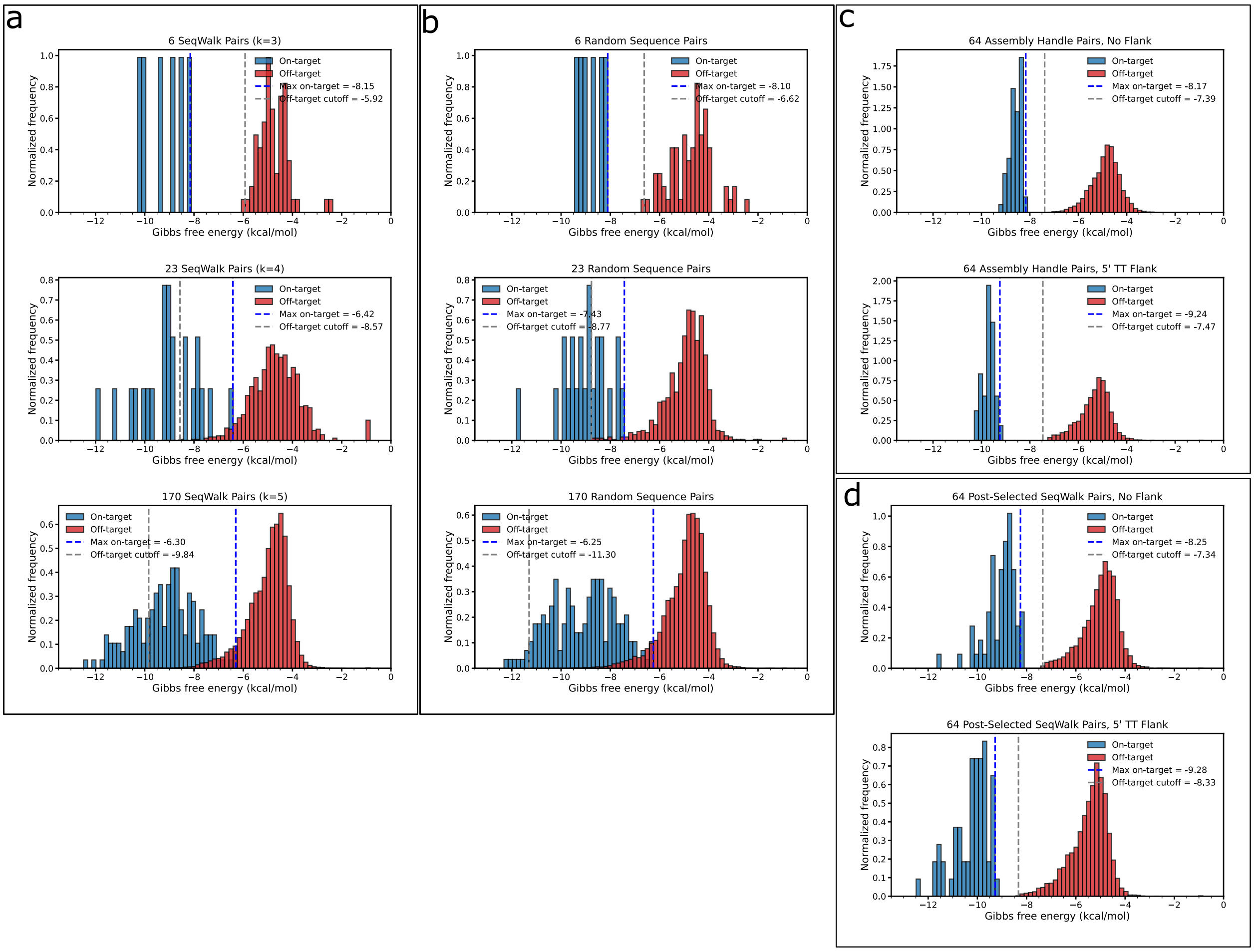

Supp. Fig. 3: Comparison of orthogonal libraries generated using seqwalk, a random selection and our sequence search strategy. **a** On-target and off-target energy distributions for seqwalk-generated sequences for $k = 3, 4, 5$. **b** Corresponding distributions for randomly generated sequence pairs with the same library size. **c** Binding energy distributions for the 64 sequences from our optimized sequence library. **d** Post-selection of the $k = 5$ seqwalk library from panel a using our orthogonal sequence search algorithm to identify 64 orthogonal pairs.

We investigated whether applying our sequence search algorithm to the 170 sequence pairs generated by seqwalk for $k = 5$ could further improve orthogonality. The results are shown in Fig. 3d. To this end, we considered two cases: seqwalk pairs with and without additional TT flanks, as our sequence library was generated using TT-flanked handles. For the flanked case, we appended 5′-TT to all seqwalk sequences. We then applied an on-target energy cutoff consistent with that used for our TT-flanked library. For the unflanked case, we observed very similar energy distributions and final off-target cutoffs, with the seqwalk pre-selected pairs showing a slightly improved off-target cutoff. However, this comparison is not entirely fair, as our library was specifically optimized for TT-flanked sequences. For the TT-flanked case, our optimized library exhibits a larger separation between on-target and off-target energies compared to the post-selected seqwalk pairs, resulting in a lower off-target cutoff for a library size of 64 sequence pairs.

Overall, the use of seqwalk as a pre-selection step does not provide a measurable advantage.

5 Supplementary Note 5: Comparing the Evolutionary Algorithm with Random Handle Array Selection

To ensure that the evolutionary algorithm we described in Fig. 6 is truly more efficient than a direct brute-force method, we ran a trial comparing the algorithm with a random handle assignment approach. Using the Sunflower, a reasonably complicated multi-layer design, as the platform for this test, we ran the algorithm for the standard 2000 generations, while also generating the same number of handle configurations randomly. Supp. Fig. 4 provides the Loss trajectory for both algorithms for the full 2000 generations. The algorithm provides a clear advantage over a random approach within less than 10 generations, after which the gap between the two approaches continues to widen. The random approach provides little-to-no-benefit over the 100,000 iterations tested, making this approach highly ineffective for even the smallest crisscross design.

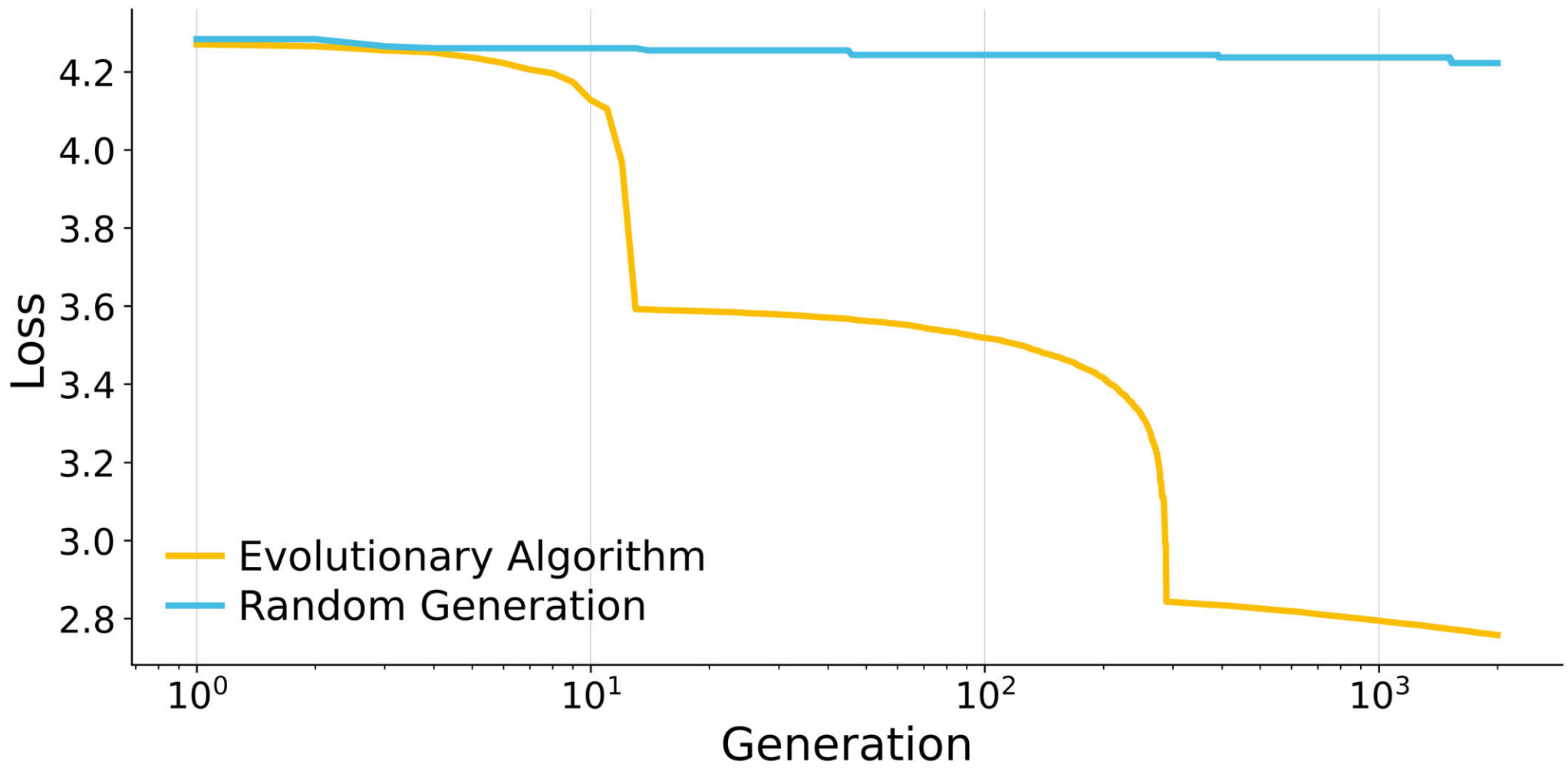

Supp. Fig. 4: Tracking the efficiency of our evolutionary algorithm and a completely random generation approach on a standard Sunflower handle assignment task (64 unique handles). For the evolutionary algorithm, we used the standard parameters of: $G = 2000, s = 3, P = 50, m = 2, pd = 0.85$. For the random approach, we generated the same number of handle arrays as the evolutionary algorithm i.e. $50 \times 2000 = 100,000$. The graph shows the best Loss for every 50 iterations of this random approach (identical to the implementation for the evolutionary algorithm).

6 Supplementary Note 6: Assembly kinetics of megastructures with different Losses

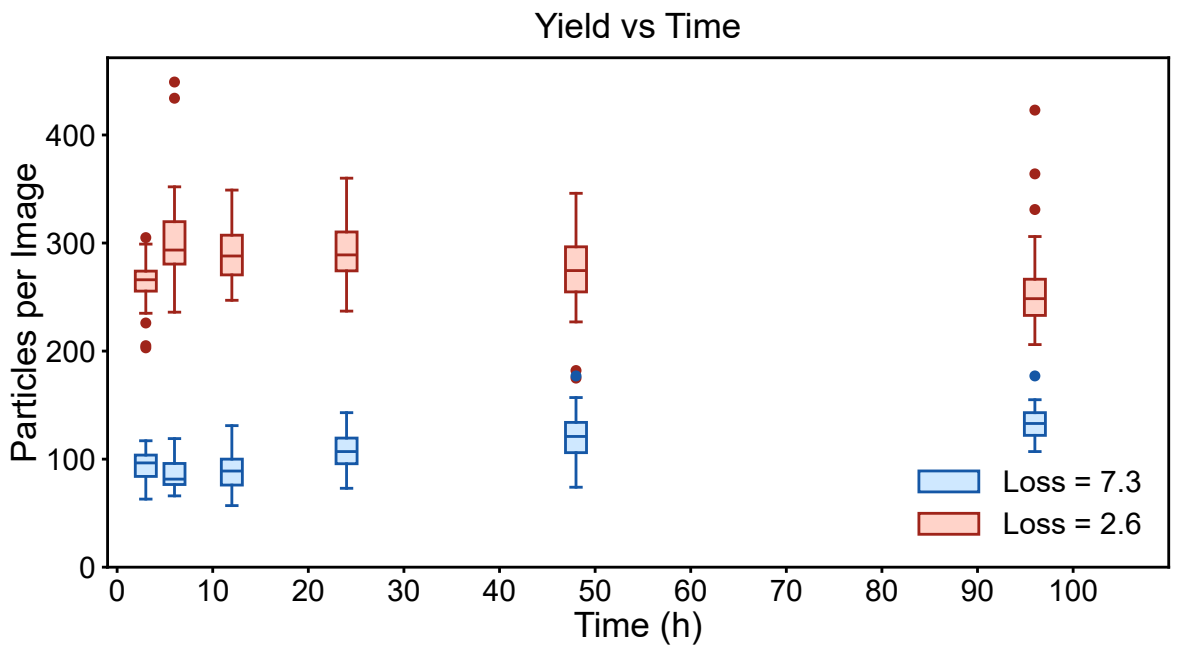

Supp. Fig. 5: **Assembly kinetics of megastructures with different Losses.** Two square megastructures with Losses of 7.3 and 2.6 were selected from the experiment shown in main text Fig. 5 and monitored over time. Assembly reactions were sampled at time points between 3 h and 96 h, while the data in the main text figure correspond to an incubation time of 41 h. For each time point and condition, we recorded 30 TEM images, counted the number of completed megastructures, and summarized the statistics in box plots. Both time traces remained constant throughout the monitored interval, with the design with higher Loss consistently yielding fewer assembled structures. This indicates that assembly in the experiment shown in main text Fig. 5 had already reached completion and that the data represent the final assembly yield. For these experiments, purification was initiated at each sampling time point by adding magnetic capture beads to the assembly mixture, allowing assembly to continue during the 15.5 h capture process. Consequently, while the data demonstrate that Loss affects the final yield, a potential influence on the assembly rate cannot be excluded (see Method 19).

7 Supplementary Note 7: Optimal parameter search of the evolutionary algorithm

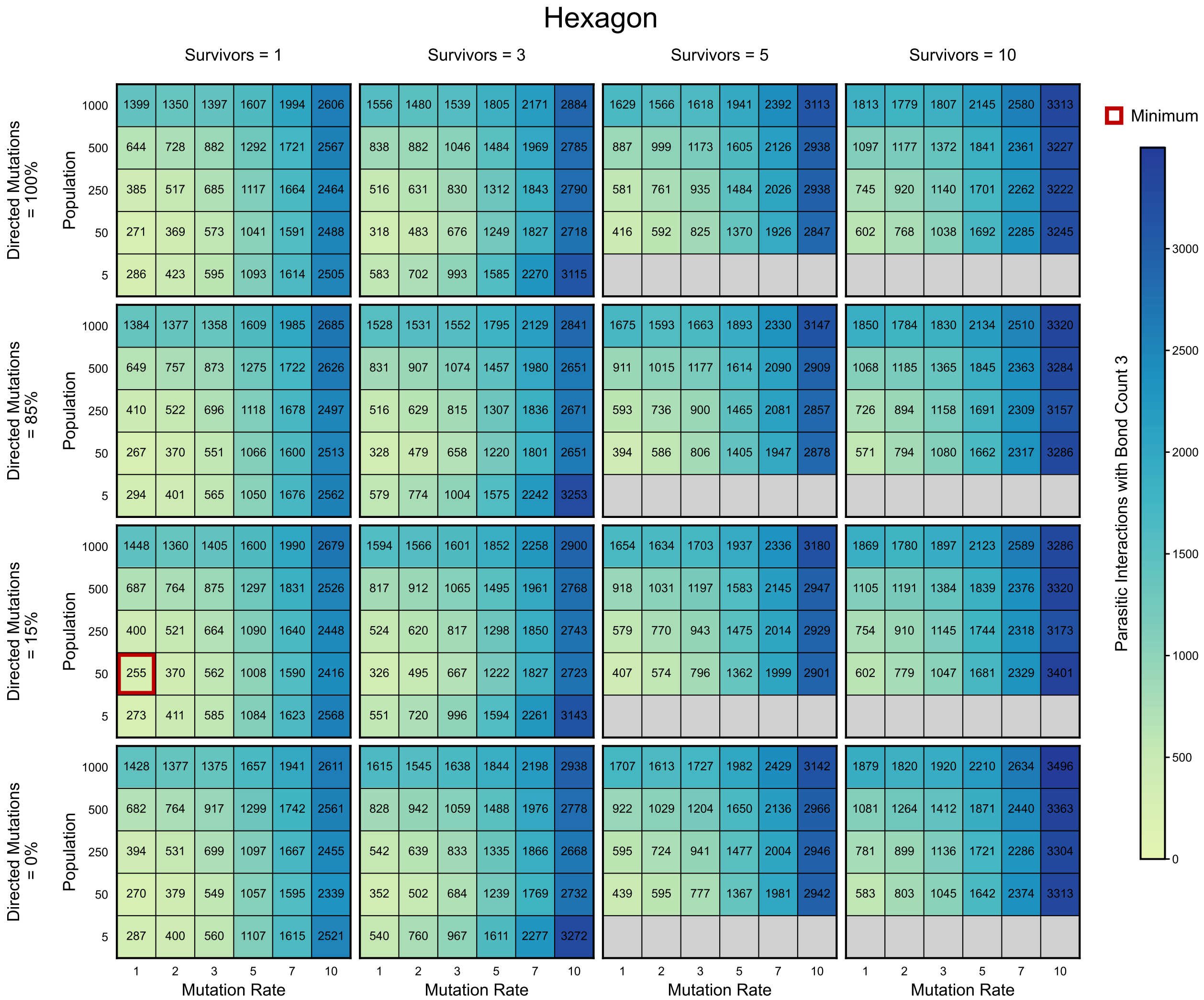

Supp. Fig. 6: **Optimal parameter search of the evolutionary algorithm on a Hexagon.** This figure shows heat maps of the final number of parasitic interactions with bond count 3 obtained when evolving handle assignments for the two-layer Hexagon megastructure, plotted as a function of the user-adjustable parameters of the evolutionary algorithm (Method 4). These parameters are: *population size*, which specifies how many new candidate assignments we create per generation; *survivors*, defined as the number of top-performing candidates that seed the next generation; *mutation rate*, given as the expected number of handle swaps applied to each survivor to generate an offspring; and *directed mutation probability*, which defines how often mutations are restricted to slats involved in parasitic interactions with the highest bond count. To compare parameter combinations on an equal computational footing, we fixed the total number of megastructure evaluations by adjusting the number of generations such that the product of population size and generations remained constant at 500,000, keeping the total number of generated assignments the same in all runs. The optimal parameter combination is highlighted in red (*mutation rate* = 1, *survivors* = 1, *population size* = 50, *directed mutation probability* = 15%). Overall, small survivor numbers, small mutation rates, and population sizes around 50 yield the lowest number of parasitic interactions with bond count 3 under the fixed-budget constraint, whereas directed mutations provide only a modest improvement with a shallow optimum near a directed mutation probability of approximately 15%. We repeated the parameter search for the multilayer Sunflower design, and the results are shown in Supp. Fig. 7. For the Sunflower, the same trends were observed: low survivor numbers, a mutation rate of one, and a directed mutation probability of 15% performed best while the optimal population size shifted to five.

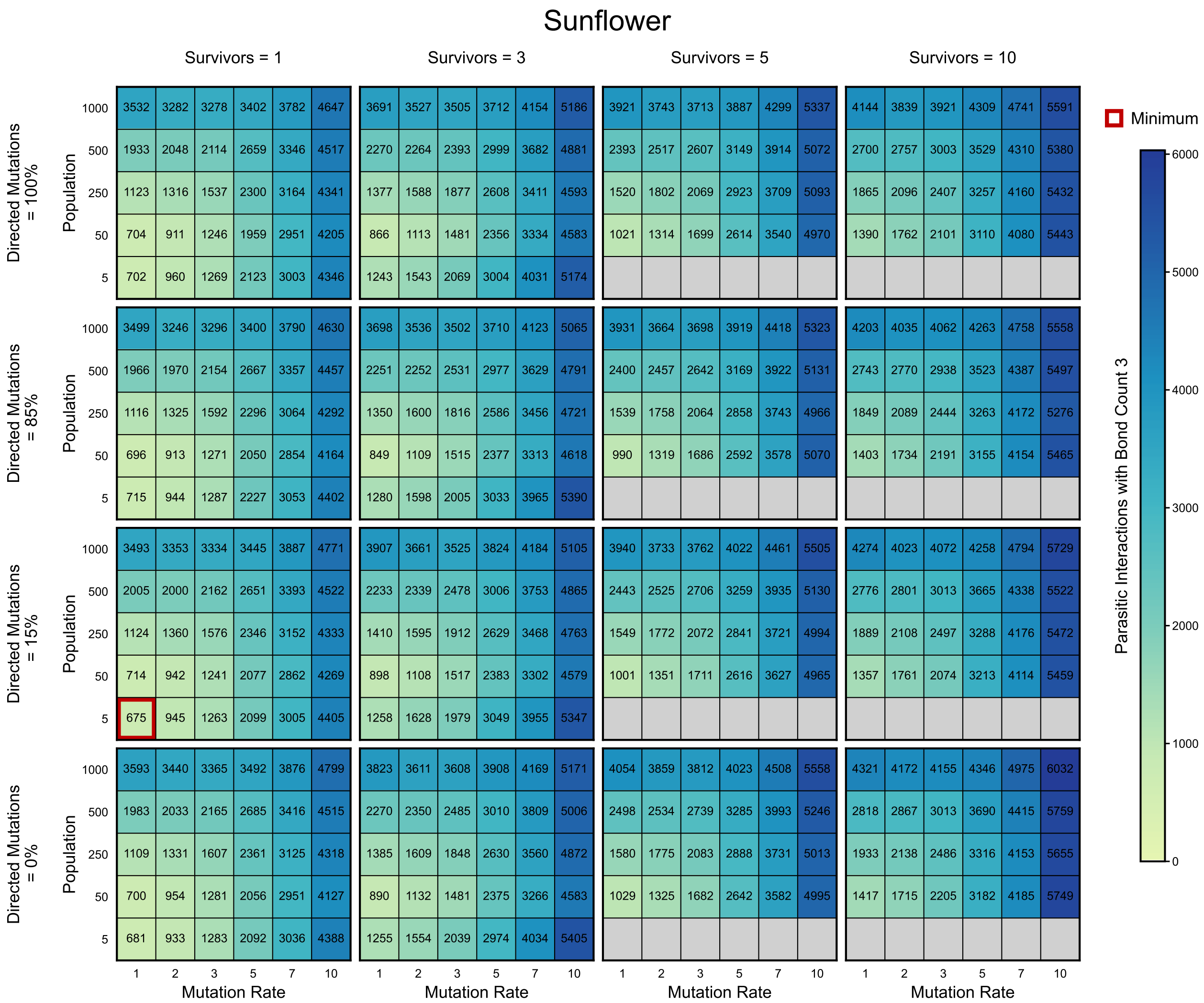

Supp. Fig. 7: **Optimal parameter search of the evolutionary algorithm on a Sunflower.** This figure shows heat maps of the final number of parasitic interactions with bond count 3 obtained when evolving handle assignments for the multilayer Sunflower megastructure. The analysis follows the same procedure as in Supp. Fig 6. Similar to the Hexagon, low survivor numbers, a mutation rate of one, and a directed mutation probability of 15% perform best. However, the optimal parameter combination differs in the population size, which shifts to five for the Sunflower geometry.

8 Supplementary Figures

The below figures contain example microscope images of the megastructures used for figures 4-7 in the main text. A much larger image dataset has been uploaded to Zenodo with DOI: https://doi.org/10.5281/zenodo.17914052. This dataset contains:

- Various images of each megastructure in Figure 4 at different magnifications.
- The entire set of images used for the quantitative results in figures 5-7.
- The data used to generate all three extended data figures.

All images in the Zenodo repository have been provided at full resolution in their raw format.

8.1 Wide Field-of-View Images of Figure 4 Megastructure Gallery

The Loss, incubation time and imaging concentration for each sample has been provided in the caption for each figure. While the concentration can give some idea of the structure yield, deposition speed on a TEM micrograph is also dependent on a particle's physical dimensions and so it is likely that each structure will deposit at a different rate.

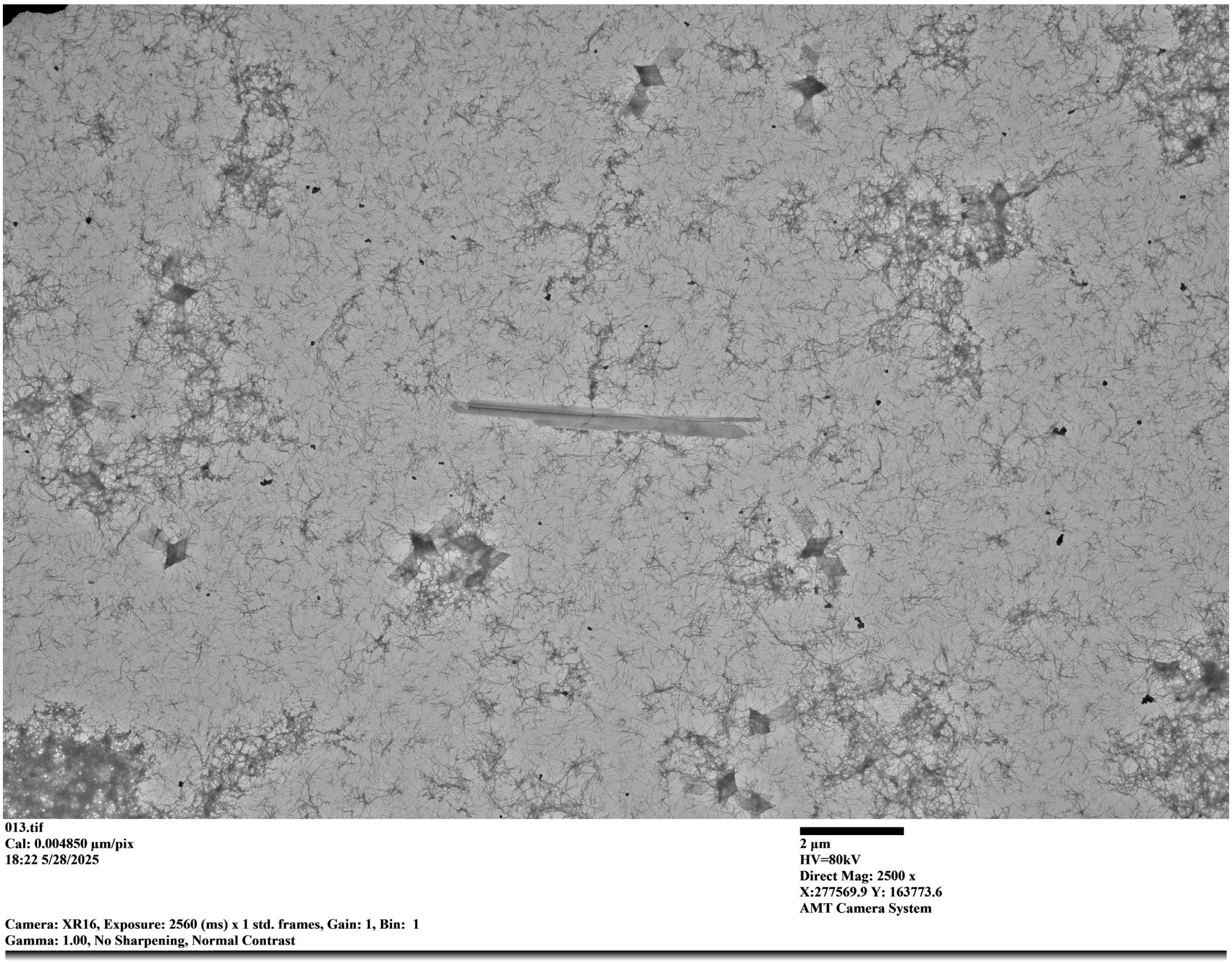

Supp. Fig. 8: Field-of-View TEM image of the 'Bird' design with a Loss of 4.46. Sample imaged at 60 pM seed concentration after 38 days of incubation. This long incubation time illustrates how megastructures can continue to assemble over long time periods while remaining stable at 37 °C.

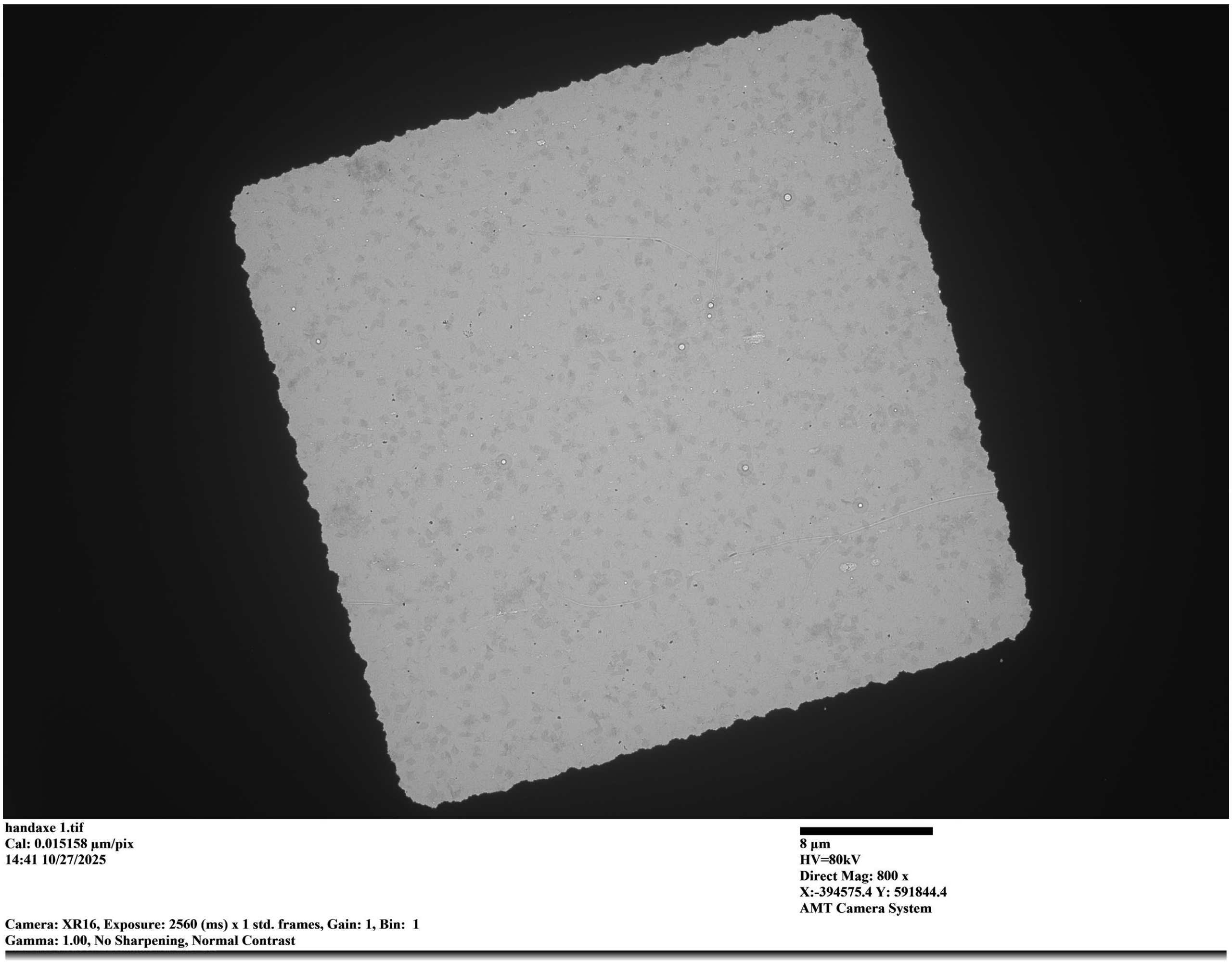

Supp. Fig. 9: Field-of-View TEM image of the 'Handaxe' design with a Loss of 2.06. Sample imaged at 500 pM seed concentration after 106 hours of incubation.

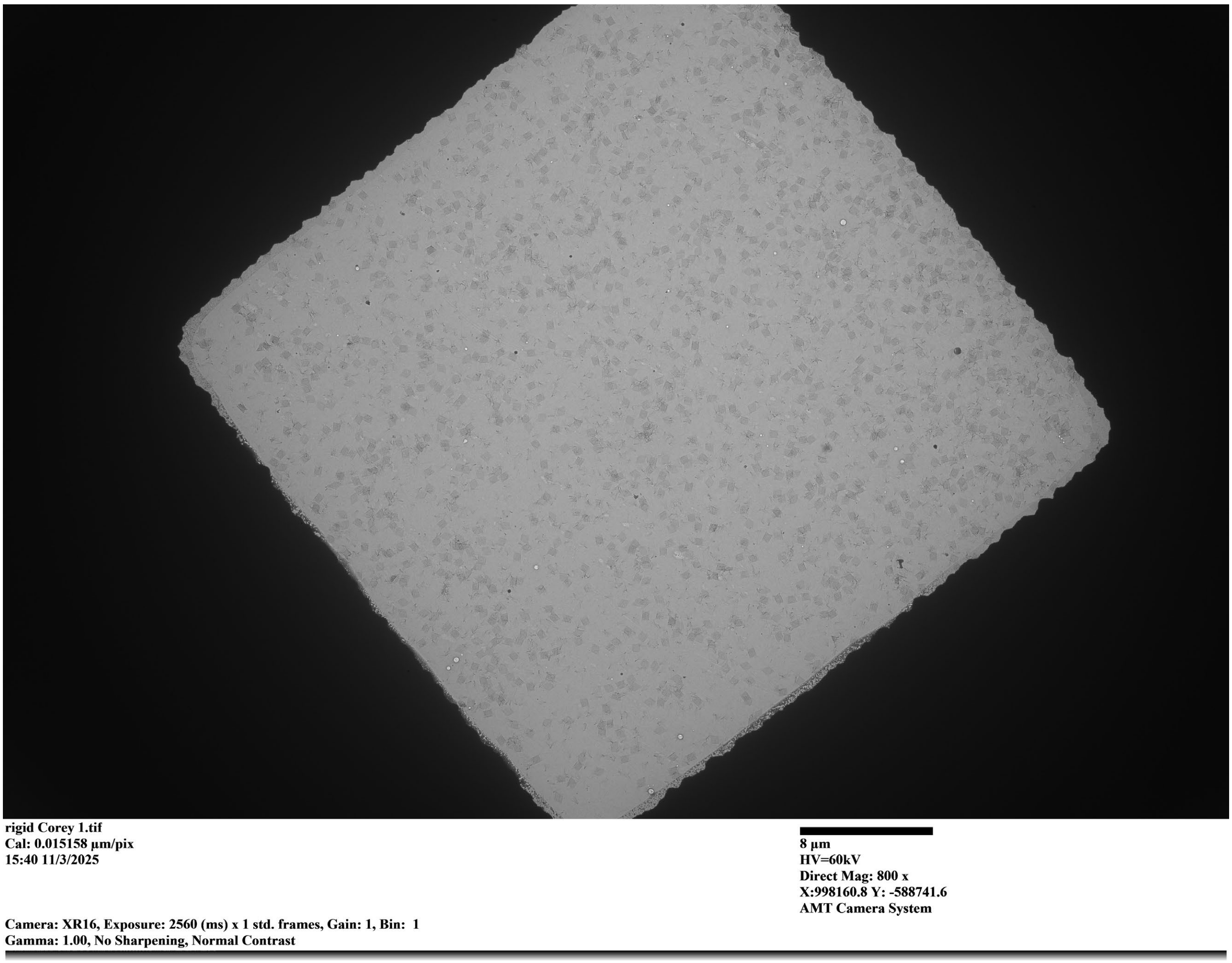

Supp. Fig. 10: Field-of-View TEM image of the 'Rigid square' design with a Loss of 2.84. Sample imaged at 123 pM seed concentration after 168 hours of incubation.

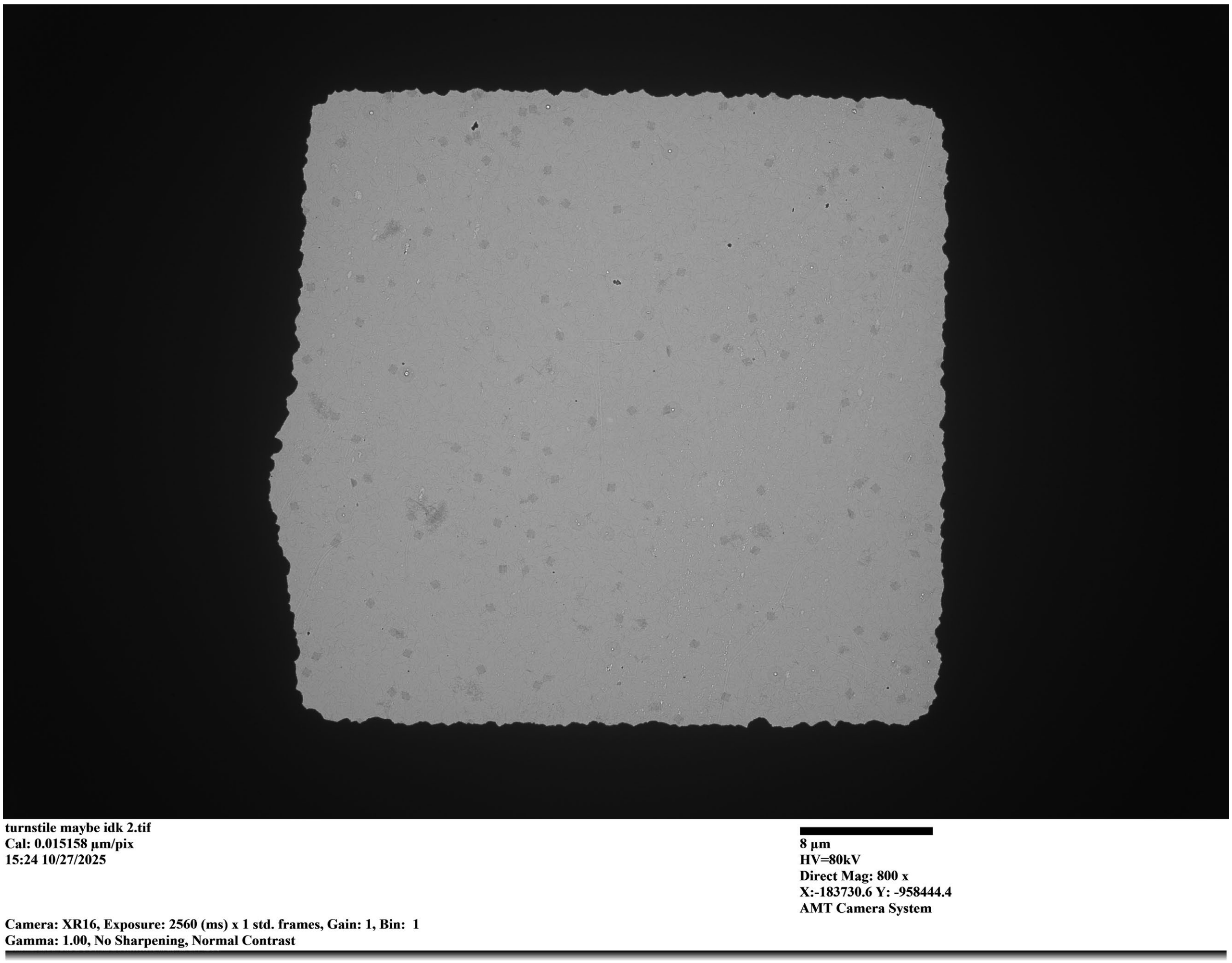

Supp. Fig. 11: Field-of-View TEM image of the 'Turnstile' design with a Loss of 2.76. Sample imaged at 250 pM seed concentration after 84 hours of incubation.

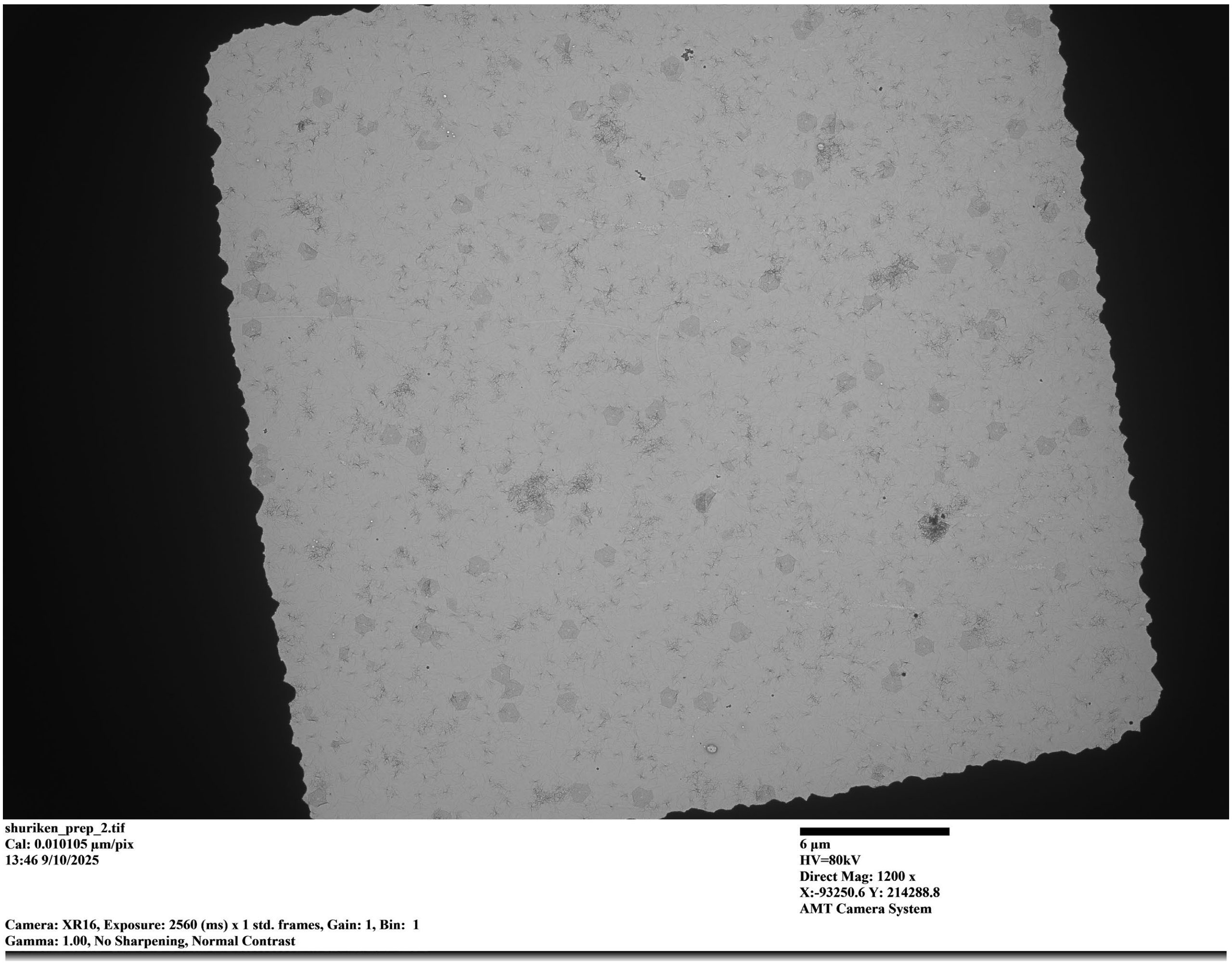

Supp. Fig. 12: Field-of-View TEM image of the ‘Shuriken’ design with a Loss of 2.7. Sample imaged at 93 pM seed concentration after 72 hours of incubation.

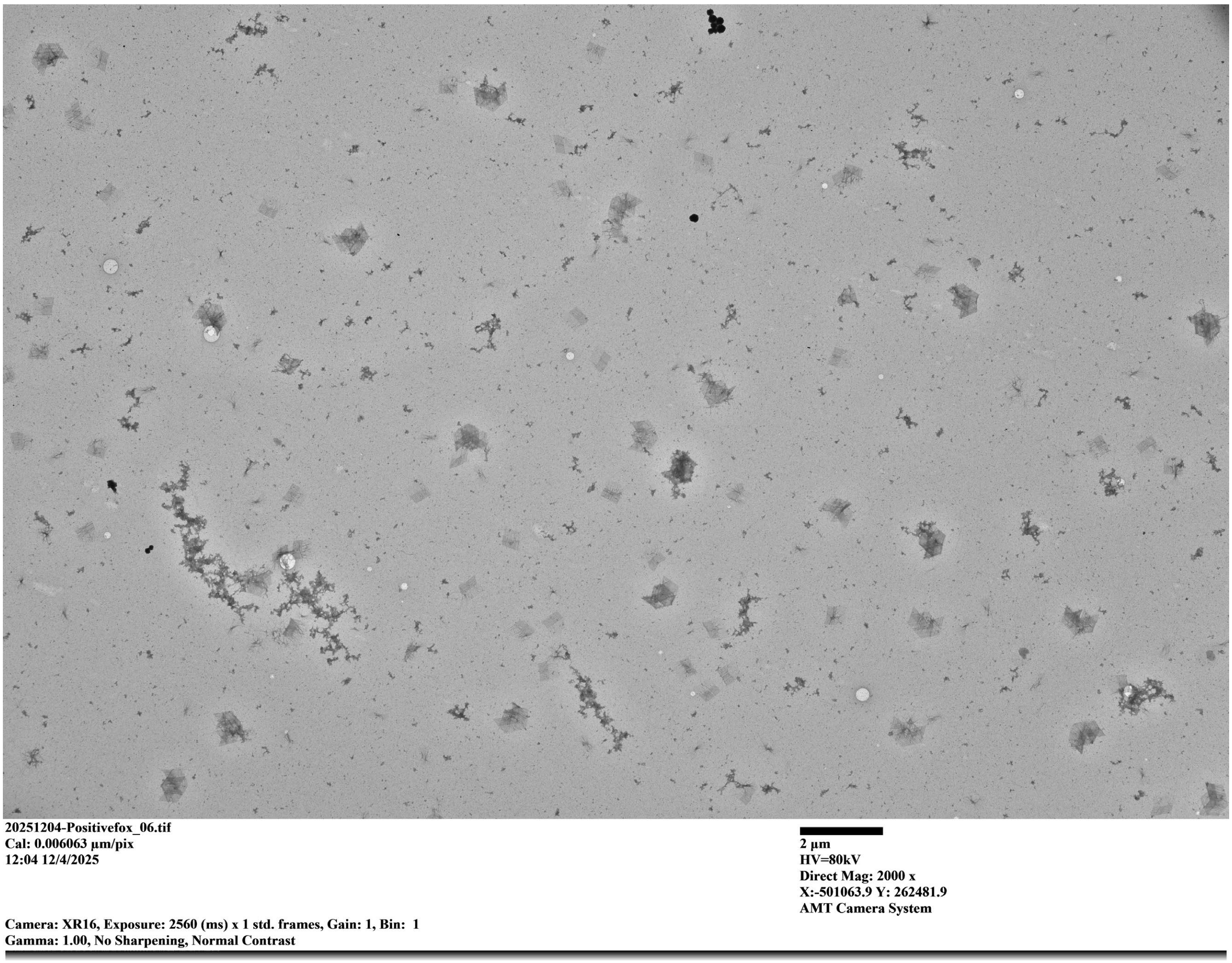

Supp. Fig. 13: Field-of-View TEM image of the ‘Fox’ design with a Loss of 2.73. Sample imaged at 70 pM seed concentration after 276 hours of incubation.

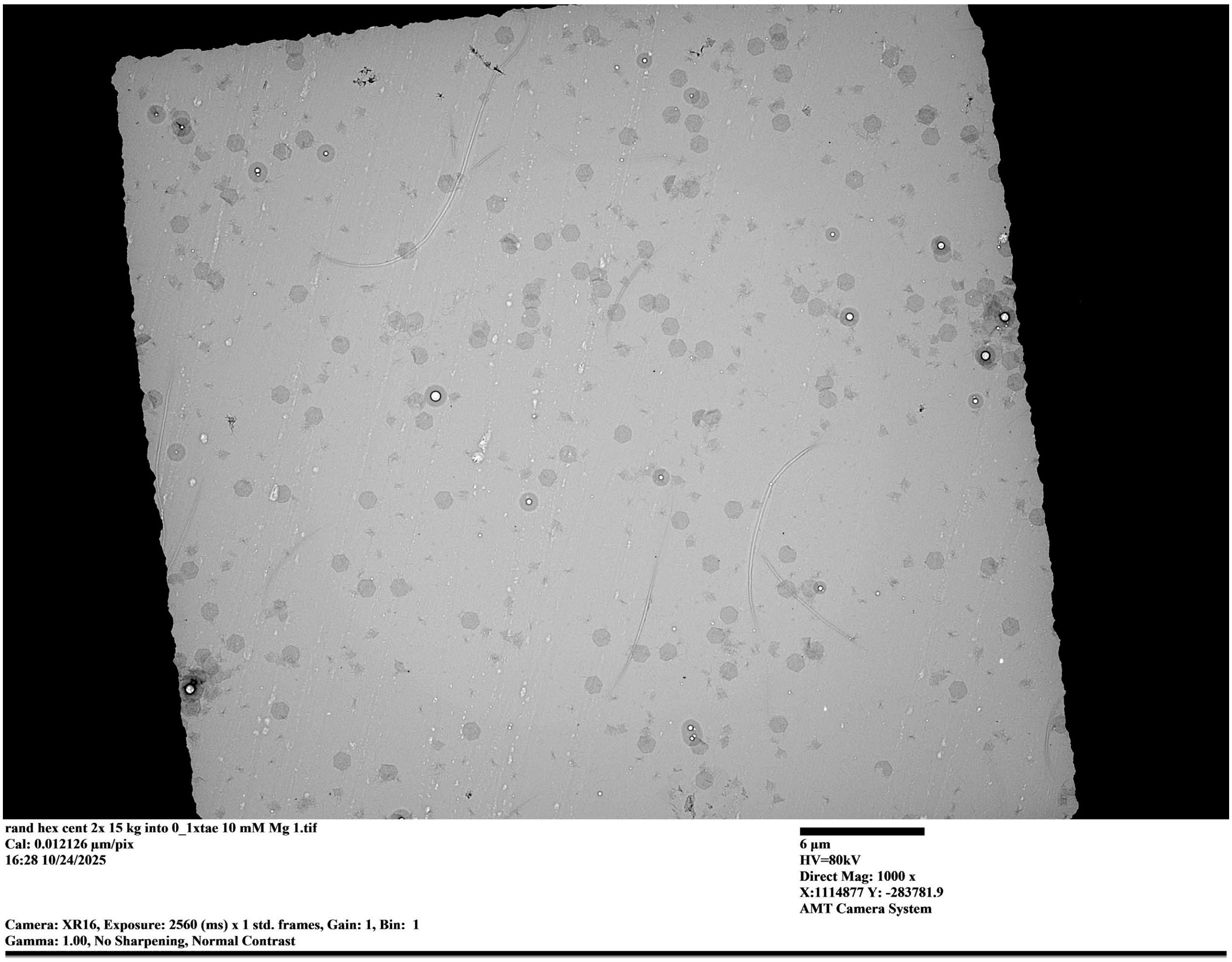

Supp. Fig. 14: Field-of-View TEM image of the ‘Hexagon’ design with a Loss of 2.7. Sample imaged at 350 pM seed concentration after 144 hours of incubation.

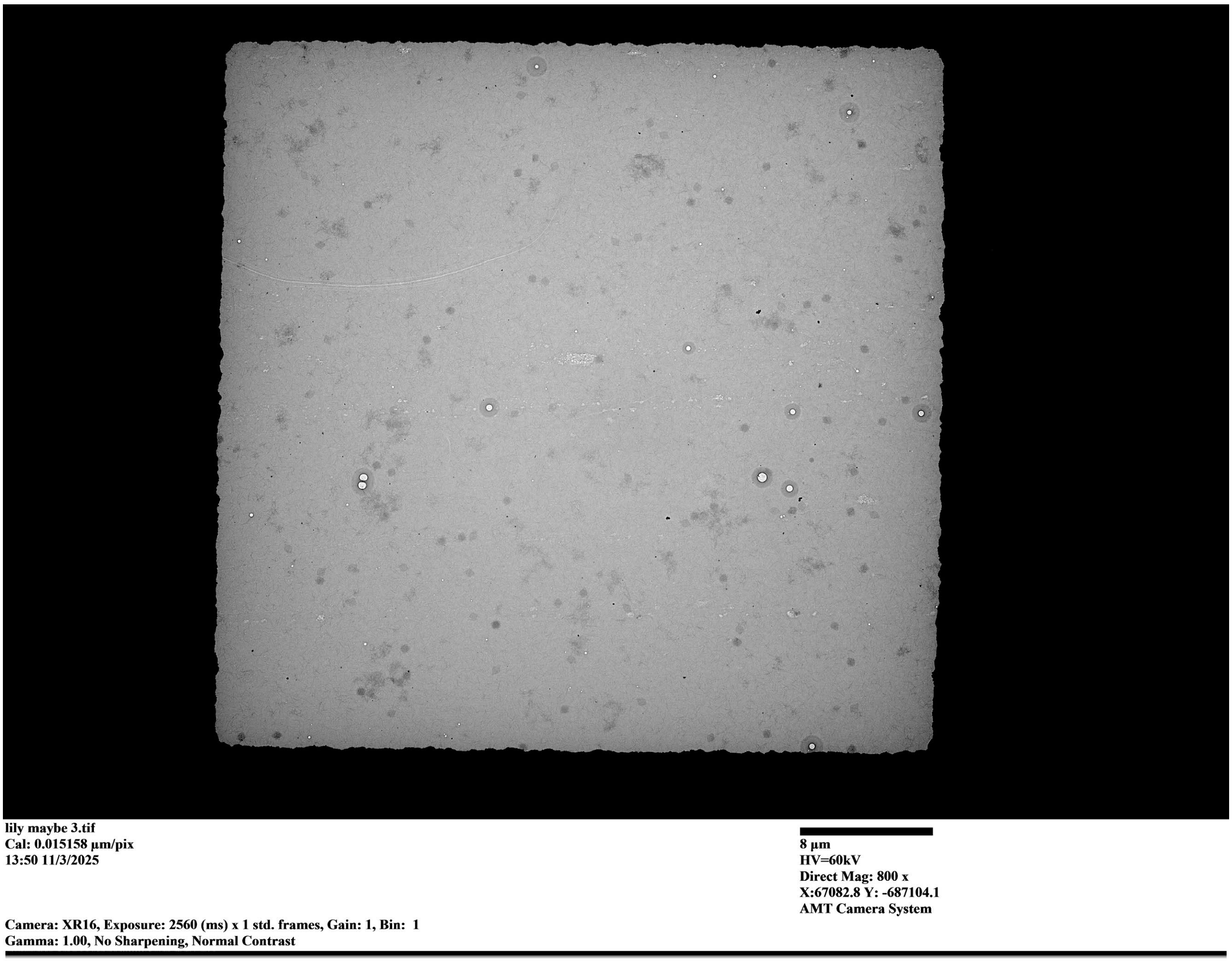

Supp. Fig. 15: Field-of-View TEM image of the 'Lily' design with a Loss of 3.8. Sample imaged at 250 pM seed concentration after 84 hours of incubation.

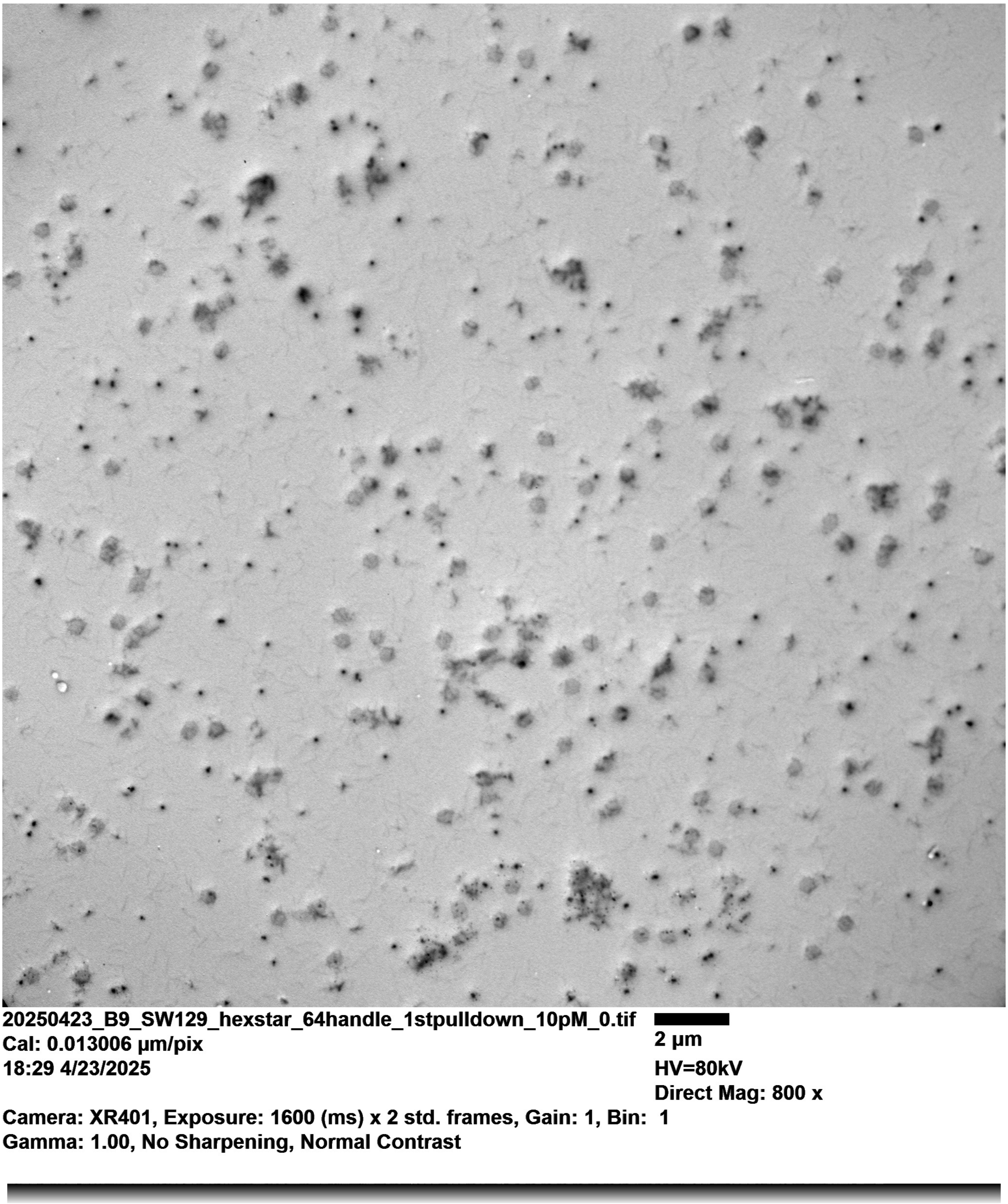

Supp. Fig. 16: Field-of-View TEM image of the 'Daffodil' design with a Loss of 2.62. Sample imaged at 10 pM seed concentration after 68 hours of incubation.

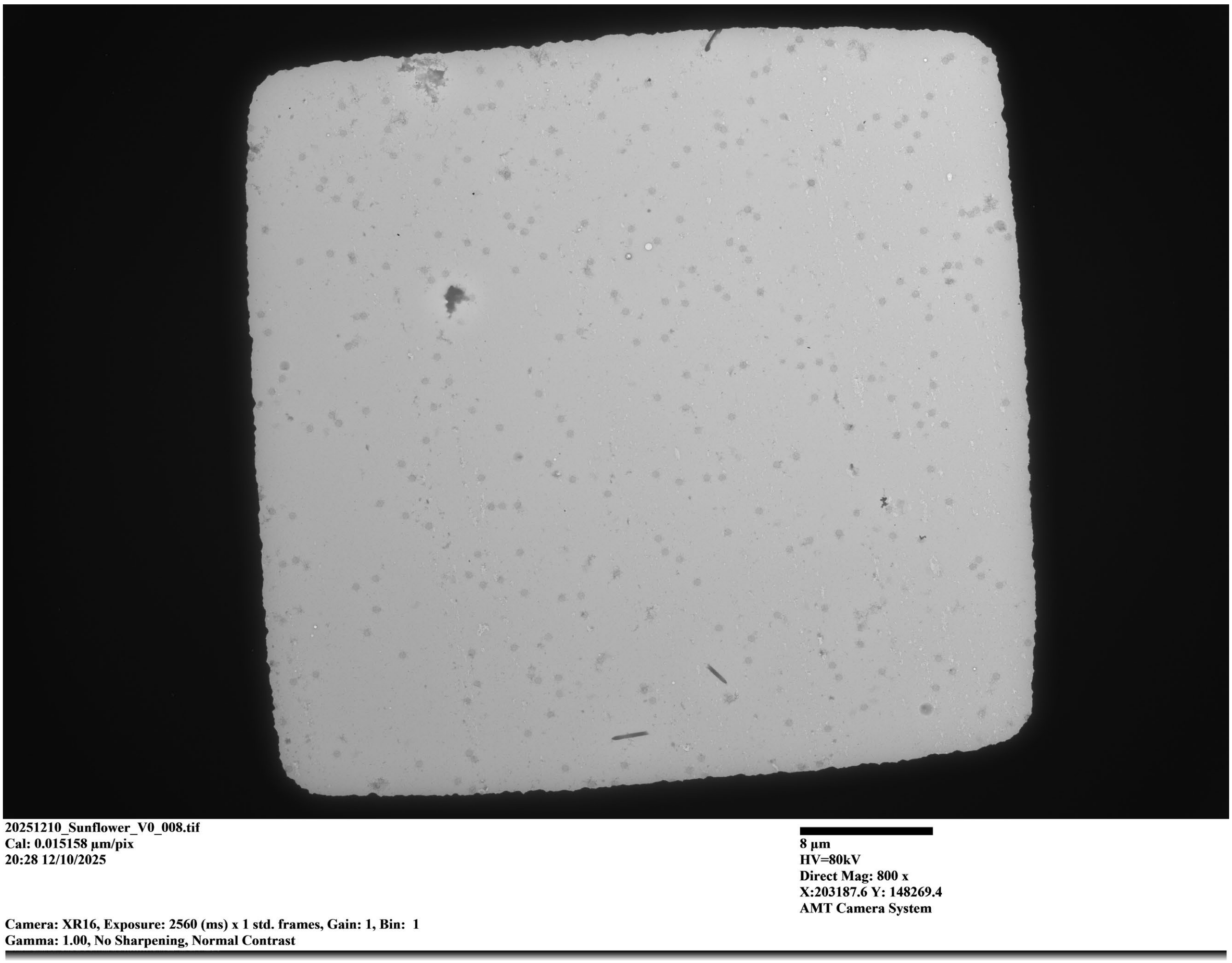

Supp. Fig. 17: Field-of-View TEM image of the 'Sunflower' design with a Loss of 3.6. Sample imaged at 130 pM seed concentration after 90 hours of incubation.

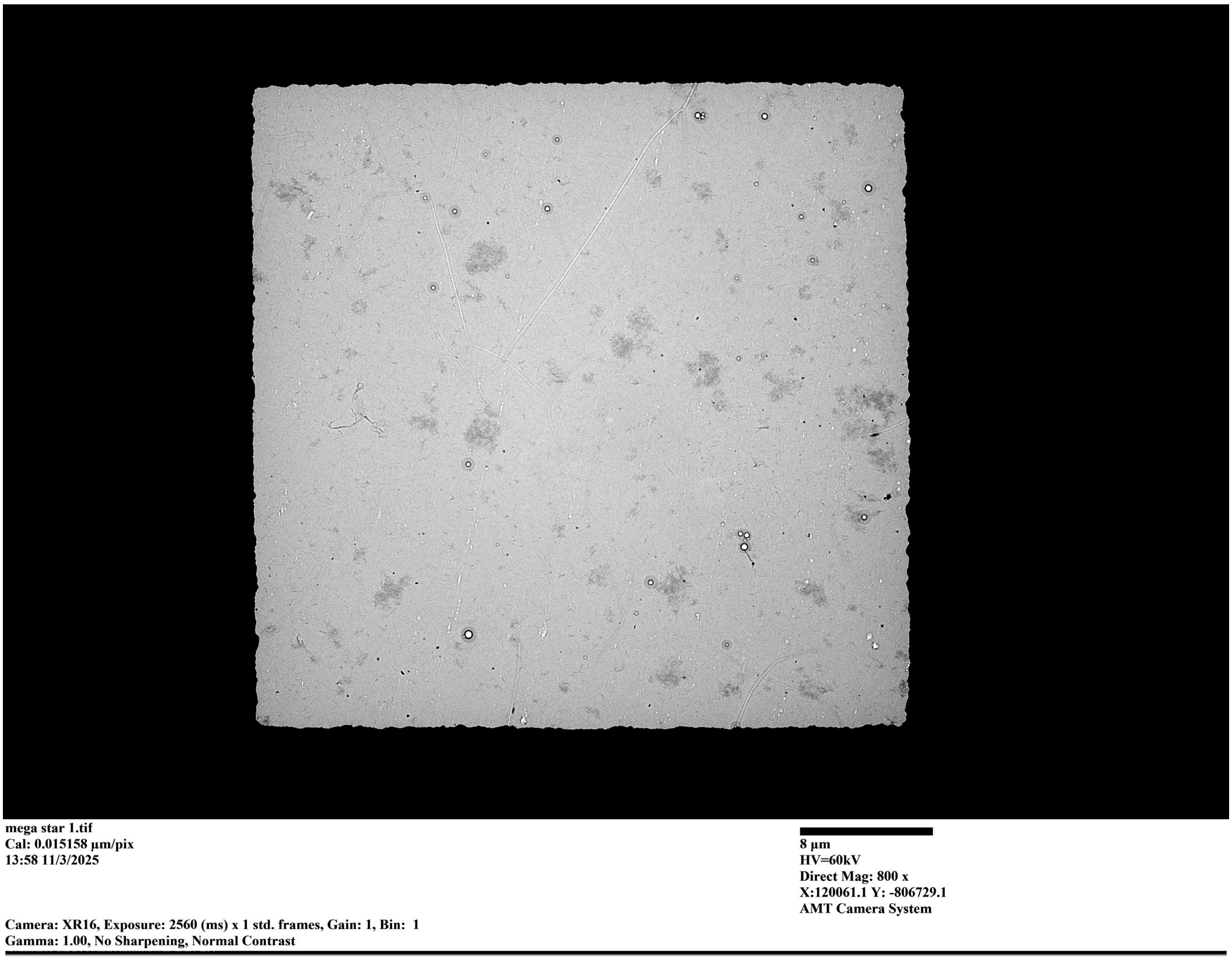

Supp. Fig. 18: Field-of-View TEM image of the ‘Megastar’ design with a Loss of 3.38. Sample imaged at 50 pM seed concentration after 106 hours of incubation.

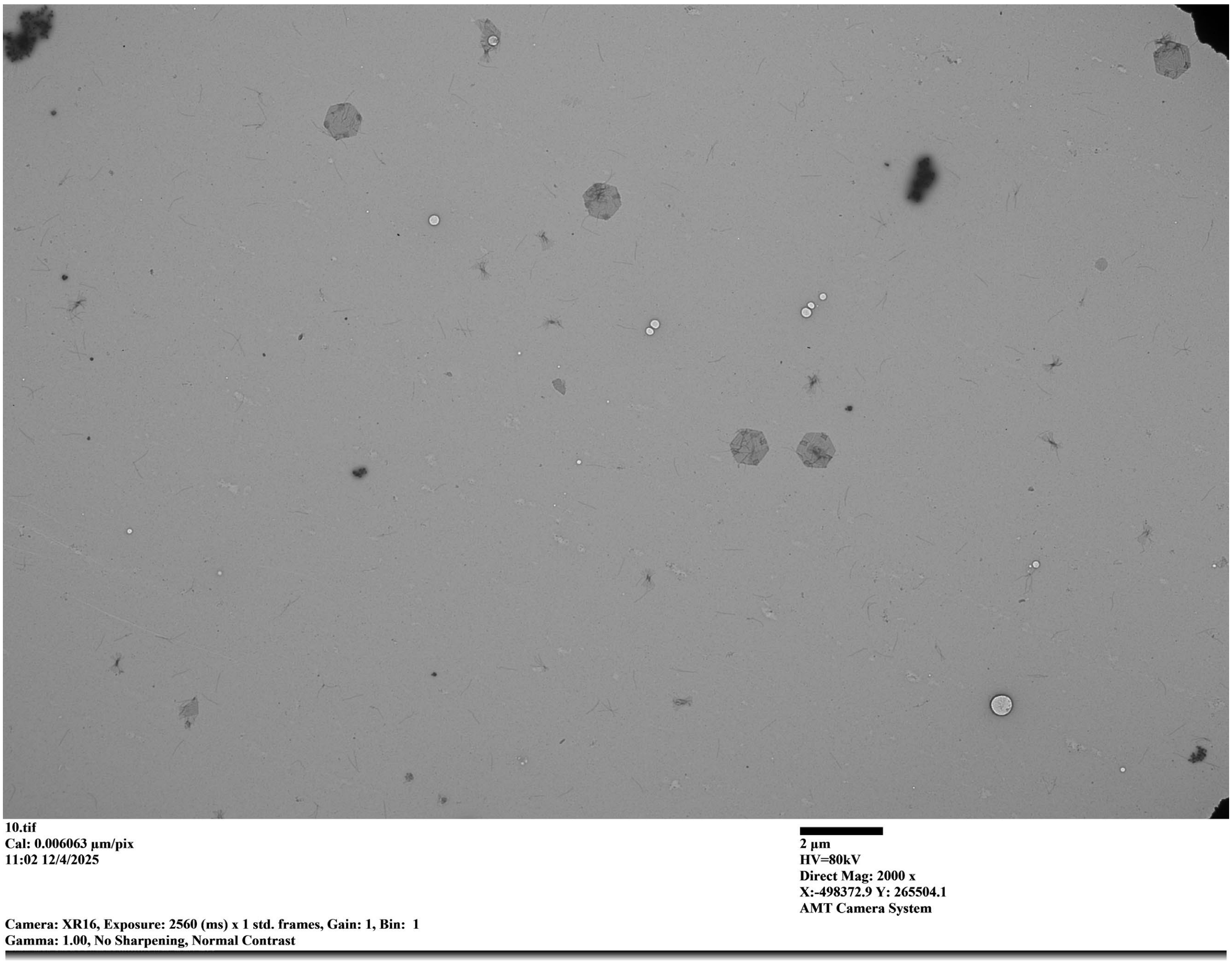

Supp. Fig. 19: Field-of-View TEM image of the ‘Hexagon’ with nanocubes attached. Sample imaged at 70 pM seed concentration after 140 hours of incubation.

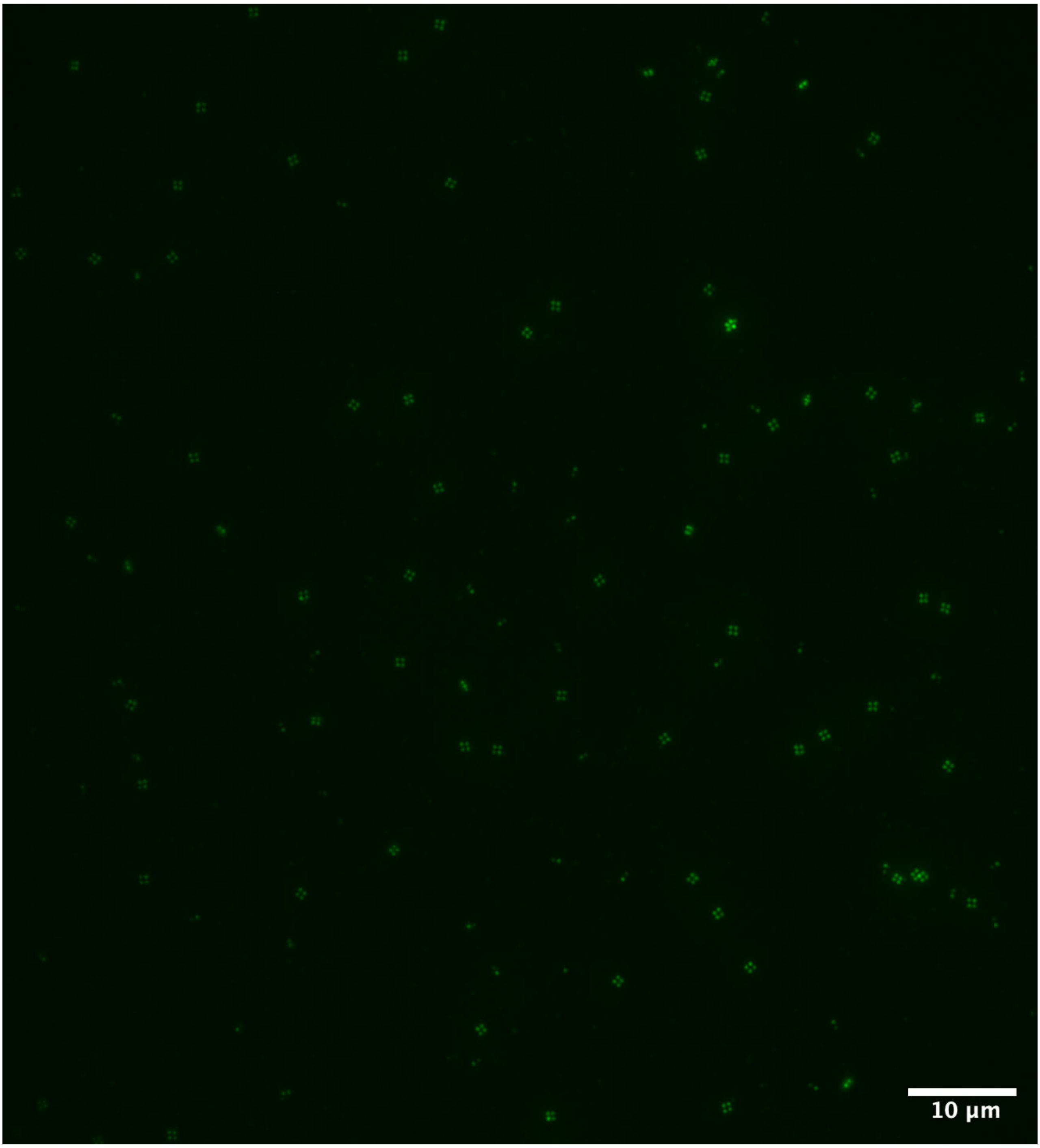

Supp. Fig. 20: Field-of-View fluorescent image of the ‘Hexagon’ with nanocubes attached (incubation time identical to that of Supp. Fig. 19).

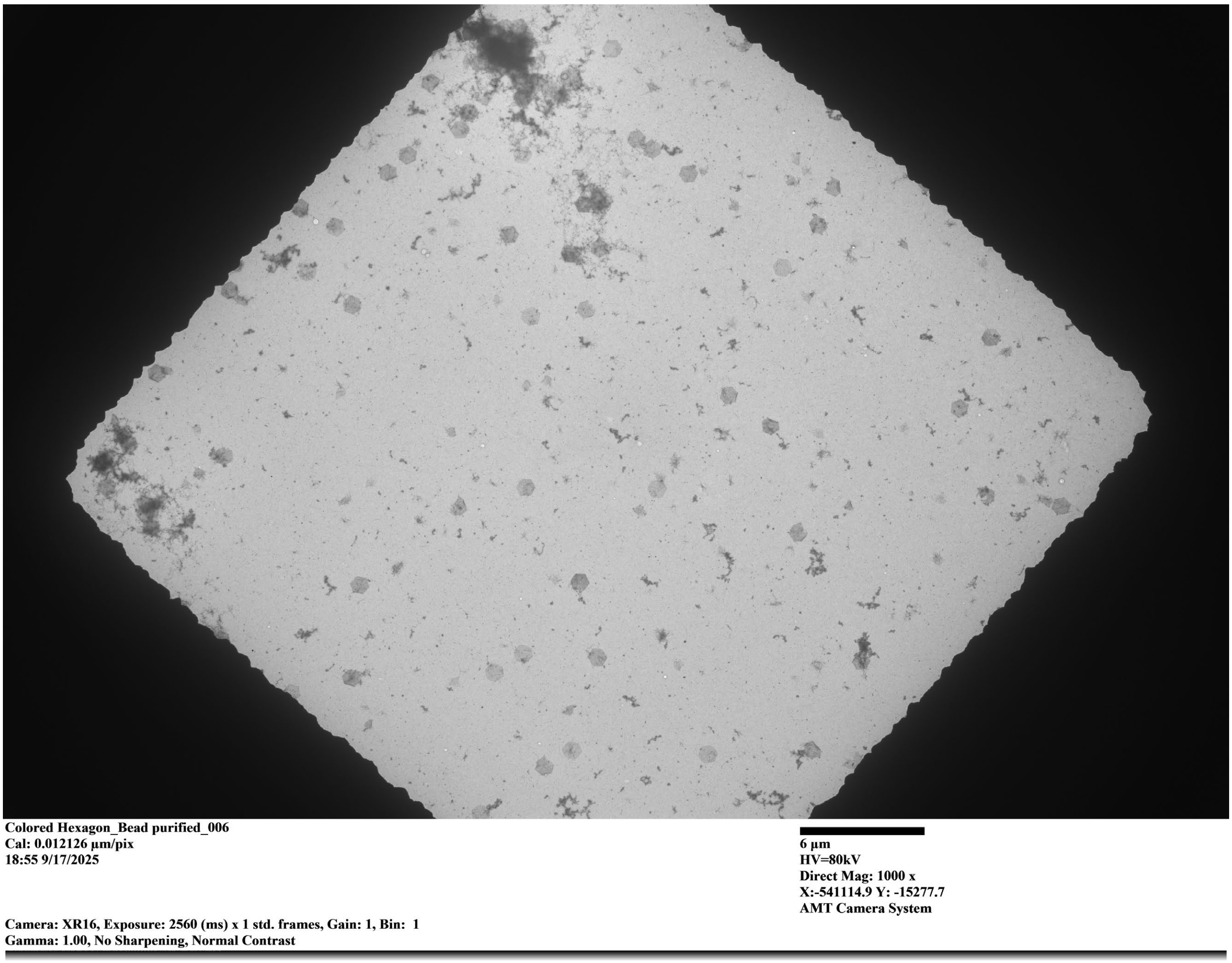

Supp. Fig. 21: Field-of-View TEM image of the 'Hexagon' with color-coded slats. Sample imaged at 70 pM seed concentration after 84 hours of incubation.

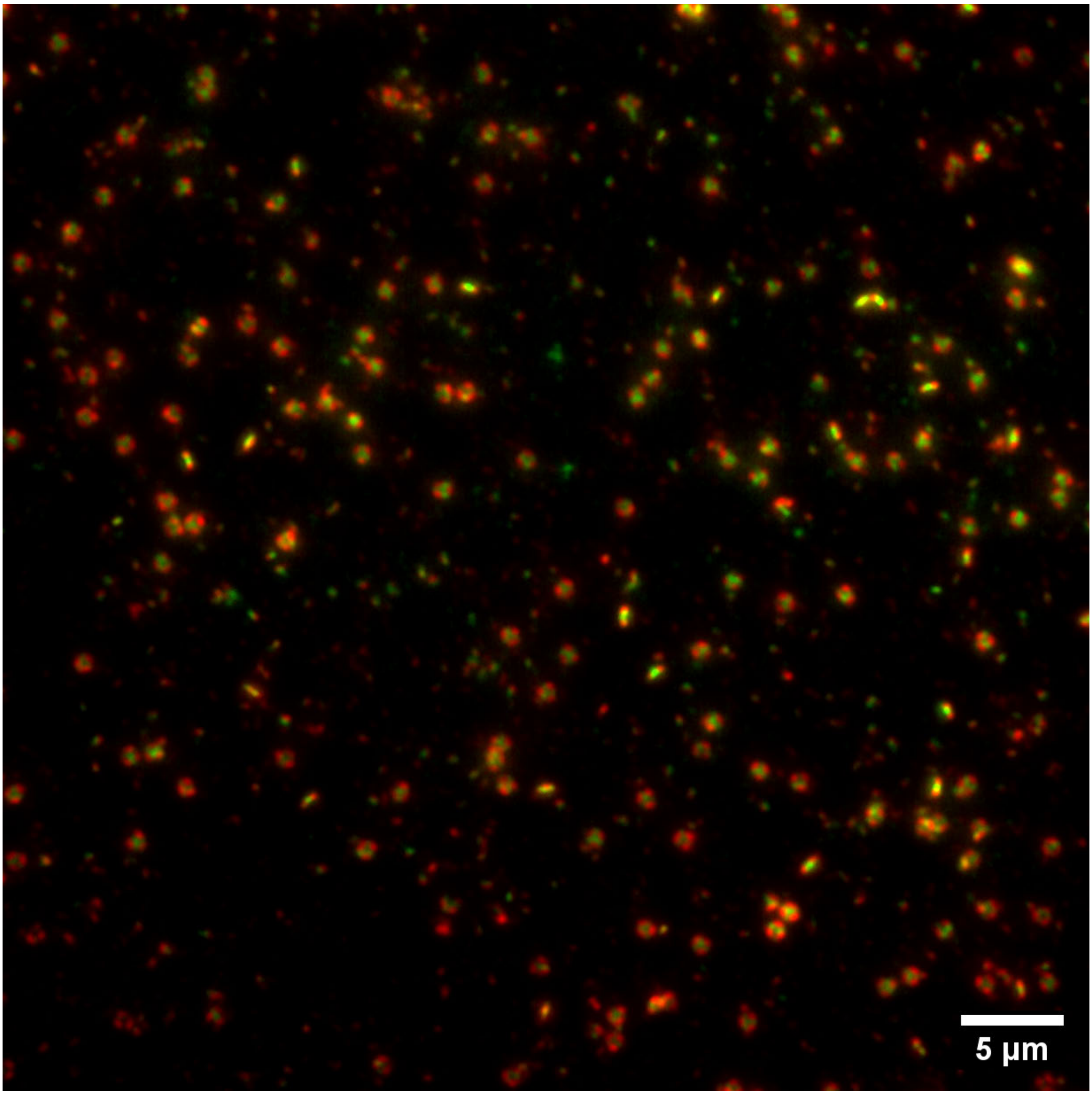

Supp. Fig. 22: Field-of-View fluorescent image of the ‘Hexagon’ with color-coded slats (incubation time identical to that of Supp. Fig. 21).

8.2 Example TEM Images from Figure 5

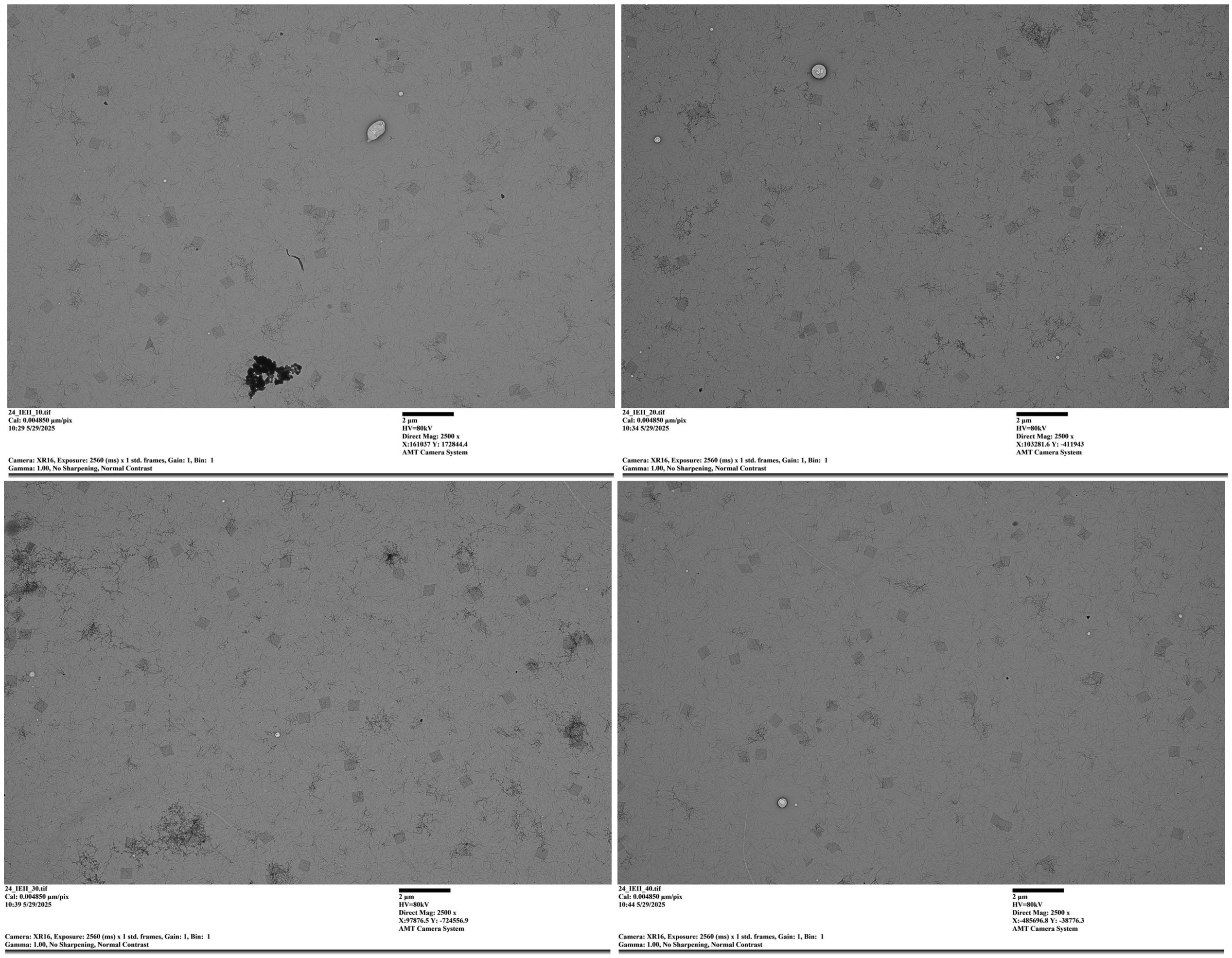

Supp. Fig. 23: Representative TEM images used to quantify the yield of the square megastructure design with **Loss = 7.3**. The corresponding yield data are shown in Figure 5.

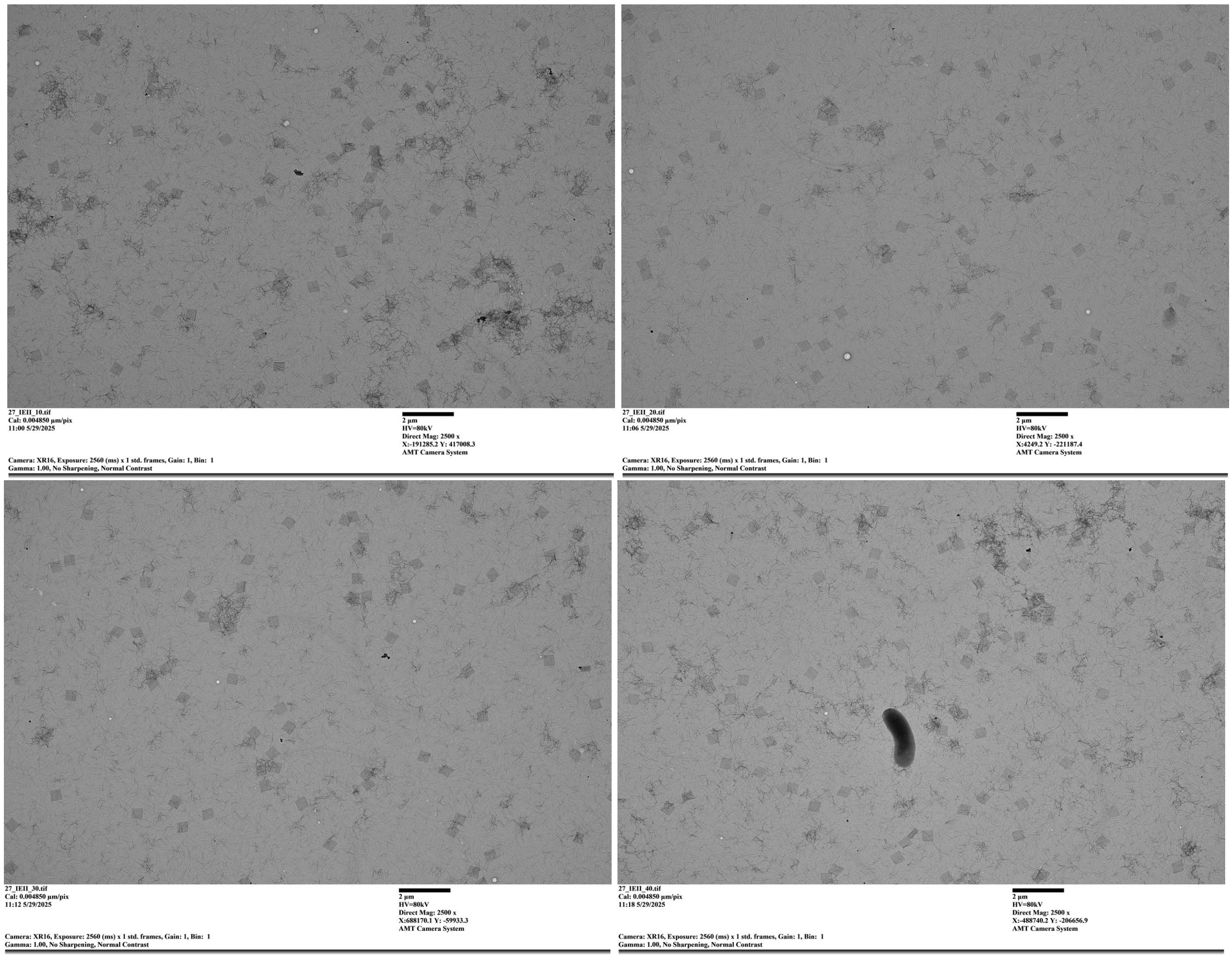

Supp. Fig. 24: Representative TEM images used to quantify the yield of the square megastructure design with **Loss = 4.7**. The corresponding yield data are shown in Figure 5.

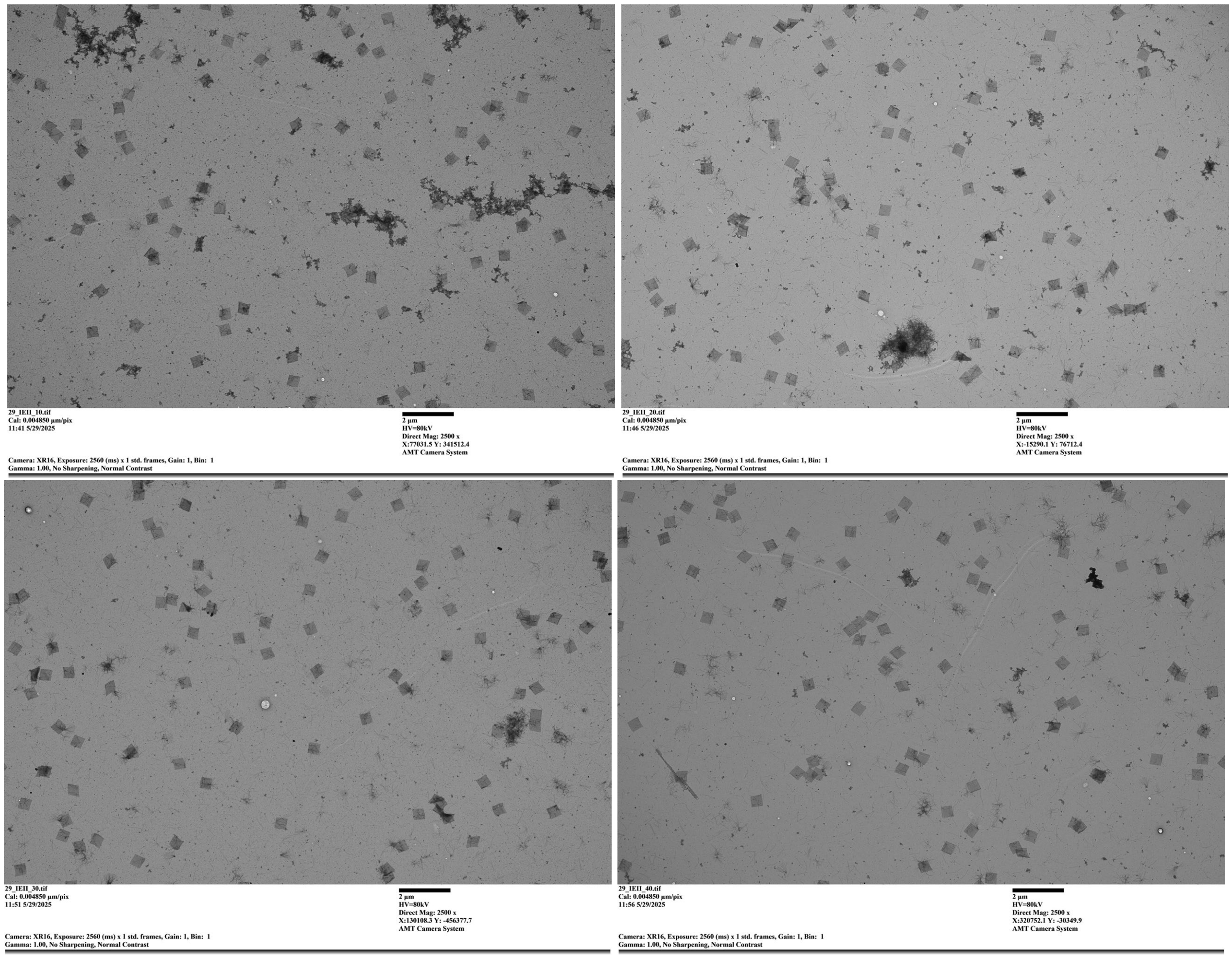

Supp. Fig. 25: Representative TEM images used to quantify the yield of the square megastructure design with **Loss = 2.6**. The corresponding yield data are shown in Figure 5.

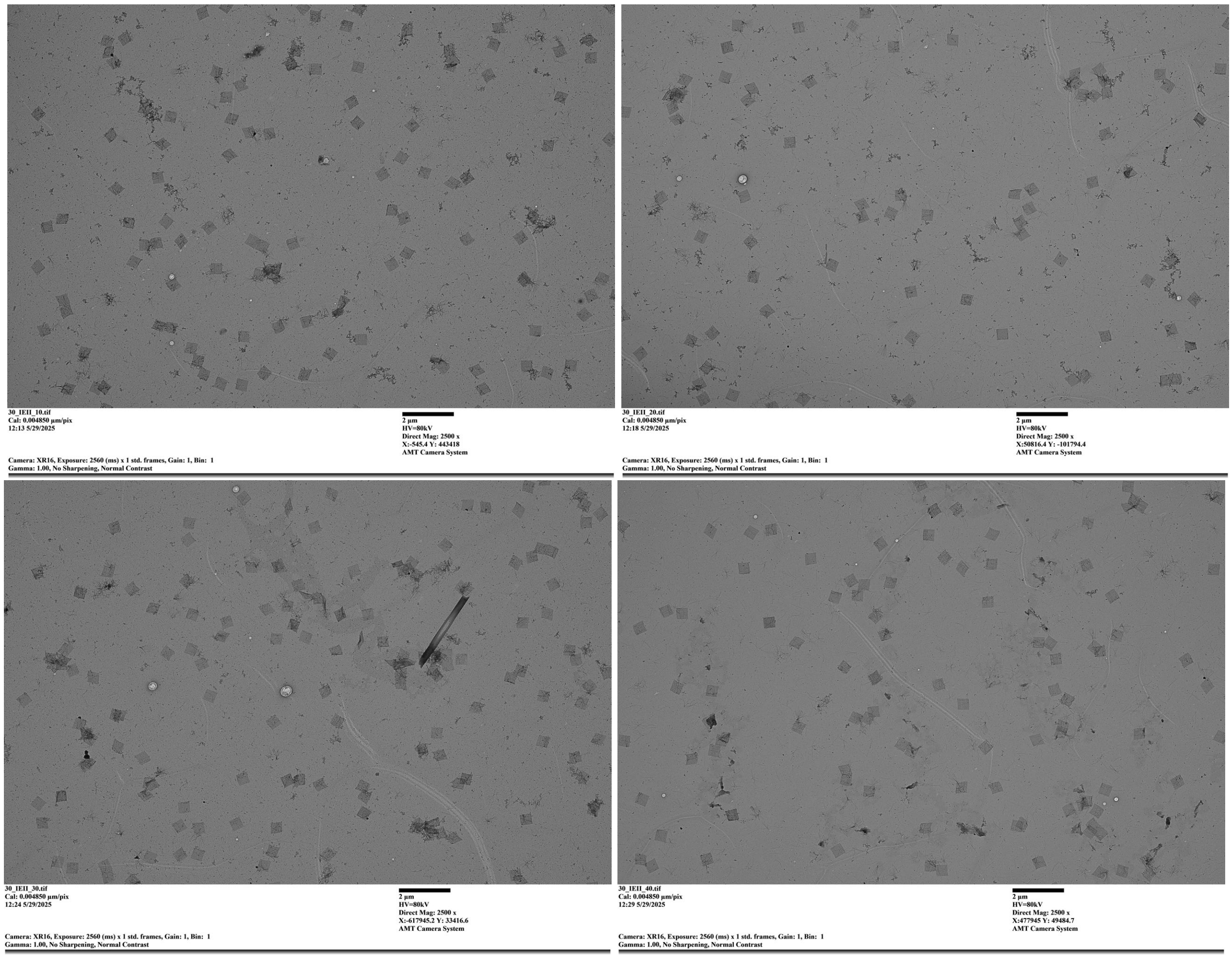

Supp. Fig. 26: Representative TEM images used to quantify the yield of the square megastructure design with **Loss = 2.1**. The corresponding yield data are shown in Figure 5 of the main text.

8.3 Example TEM Images from Figure 6/7 (sunflowers)

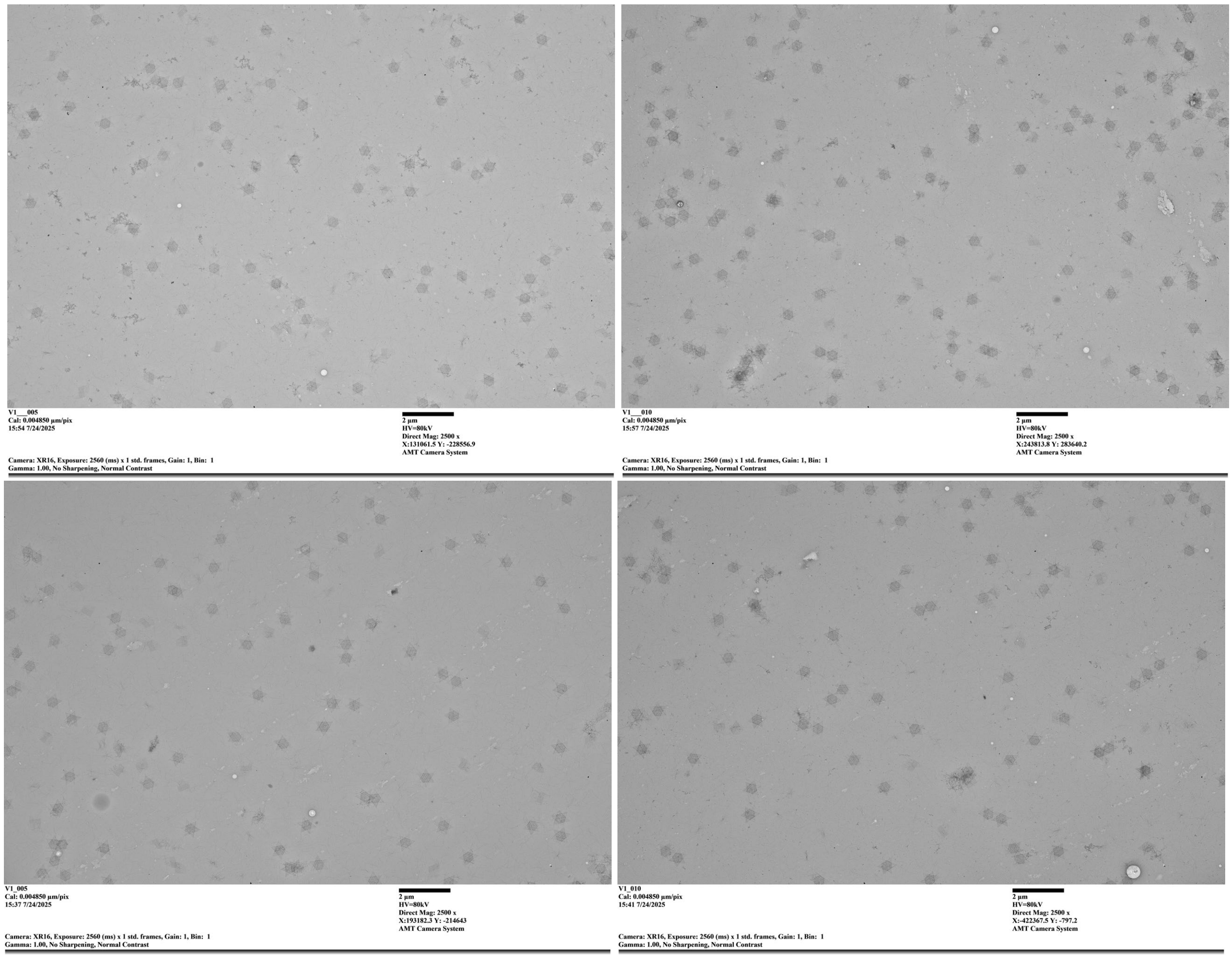

Supp. Fig. 27: Representative TEM images used to quantify the yield and structural completeness of the Sunflower megastructure design with **Loss = 2.8** using the **new** handle library. The corresponding yield data are shown in Figure 6 of the main text.

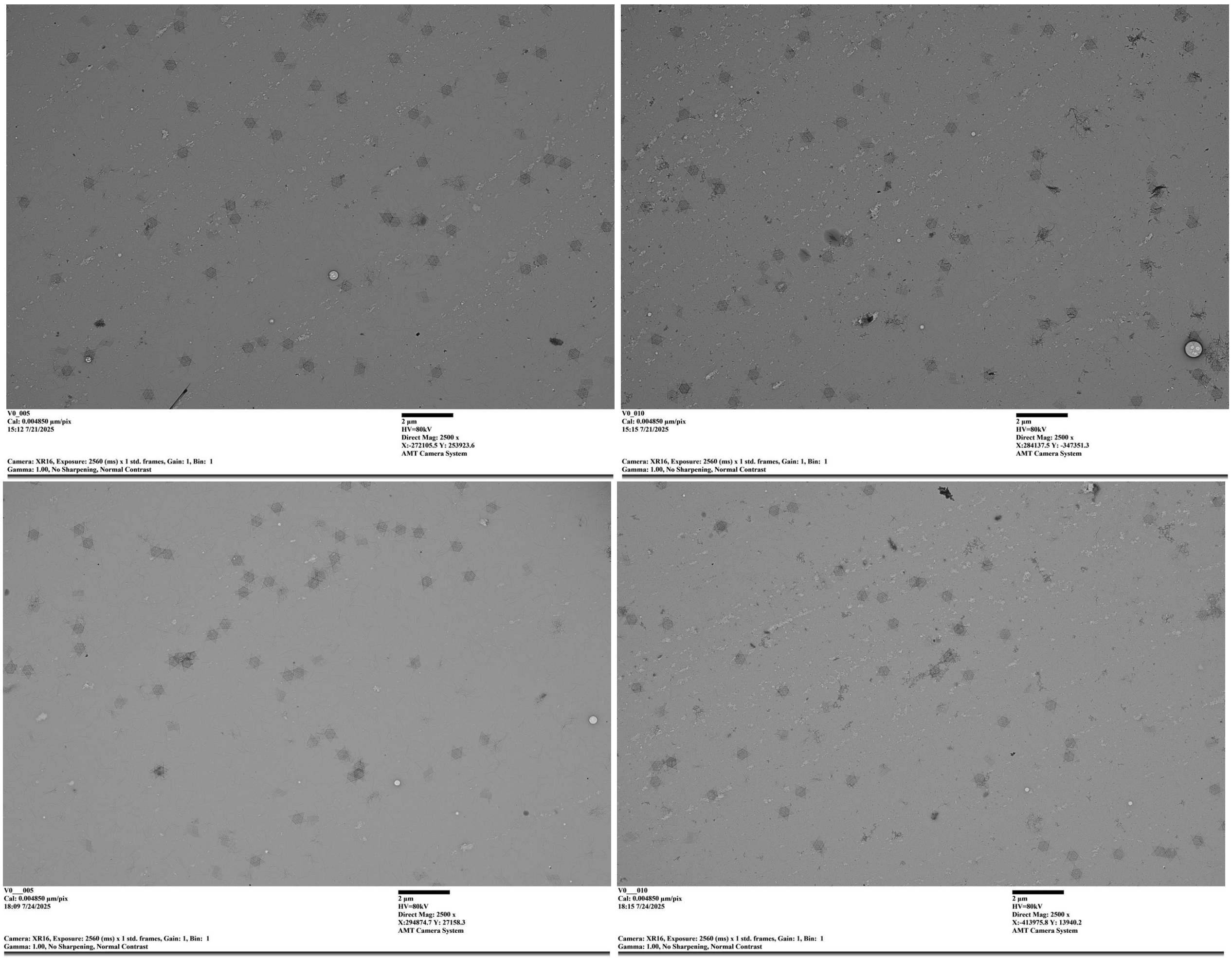

Supp. Fig. 28: Representative TEM images used to quantify the yield and structural completeness of the Sunflower megastructure design with **Loss = 3.6** using the **new** handle library. The corresponding yield data are shown in Figure 6 of the main text.

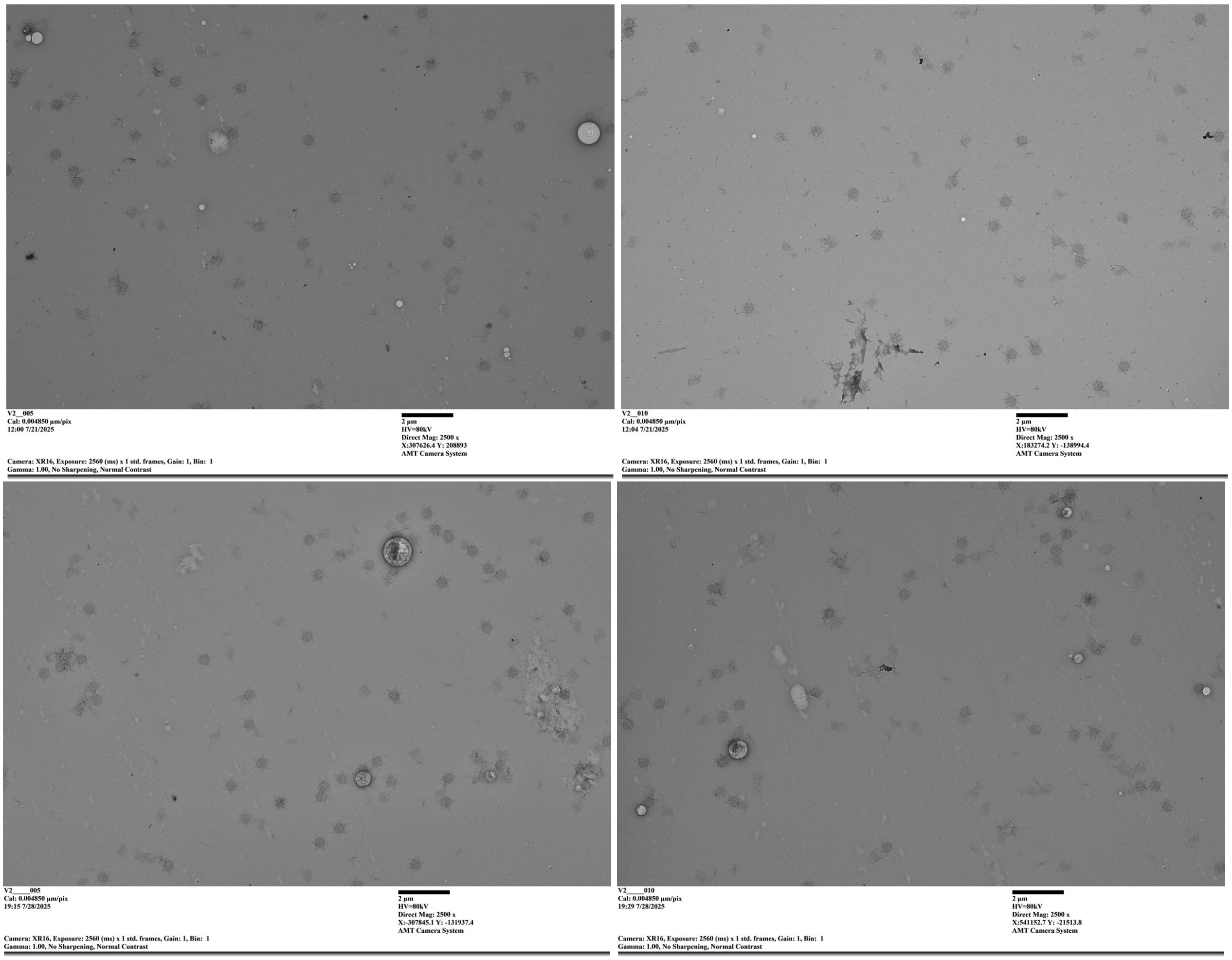

Supp. Fig. 29: Representative TEM images used to quantify the yield and structural completeness of the Sunflower megastructure design with **Loss = 3.8** using the **new** handle library. The corresponding yield data are shown in Figure 6 of the main text.

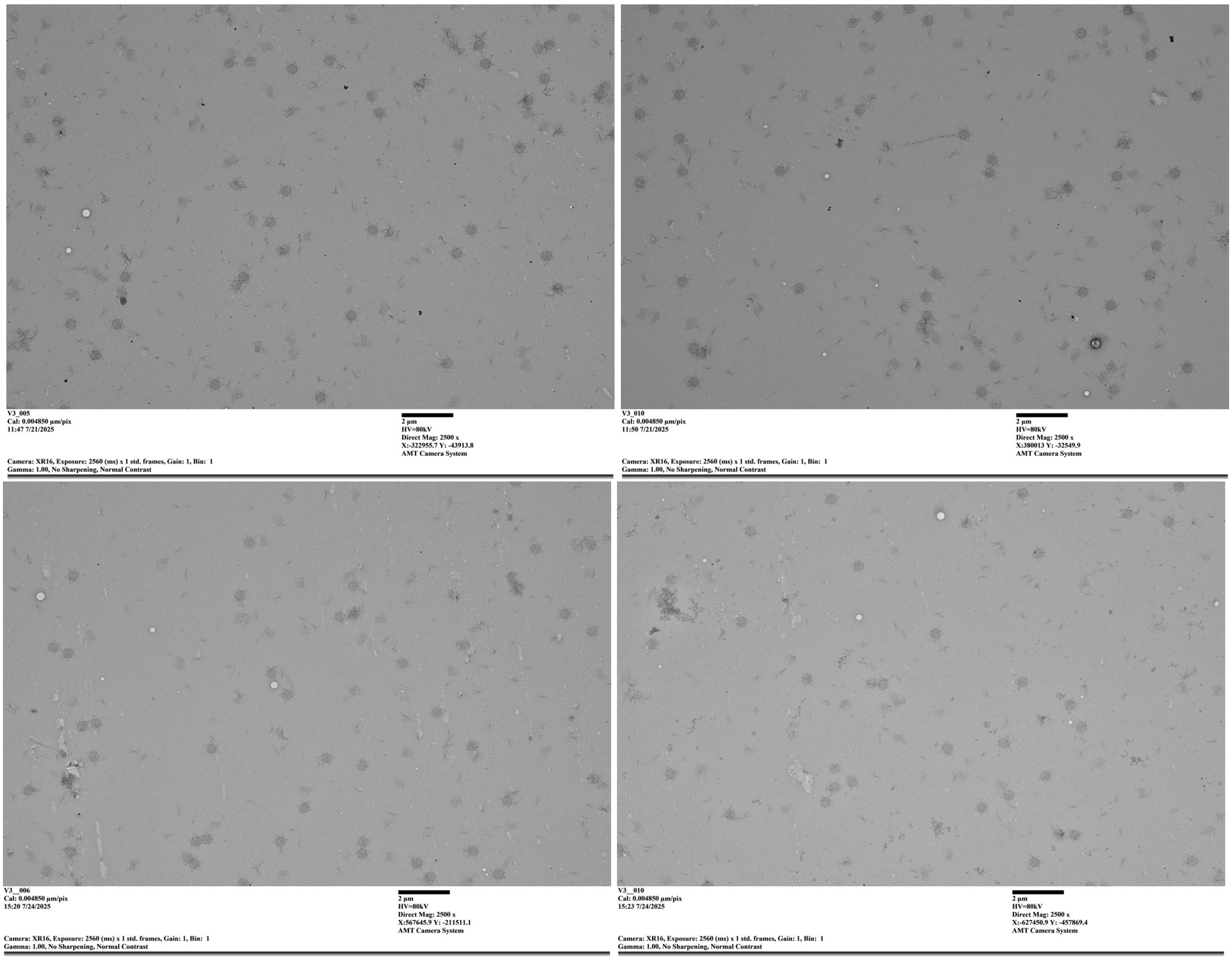

Supp. Fig. 30: Representative TEM images used to quantify the yield and structural completeness of the Sunflower megastructure design with **Loss = 3.8** using the **old** handle library. The corresponding yield data are shown in Figure 7 of the main text.

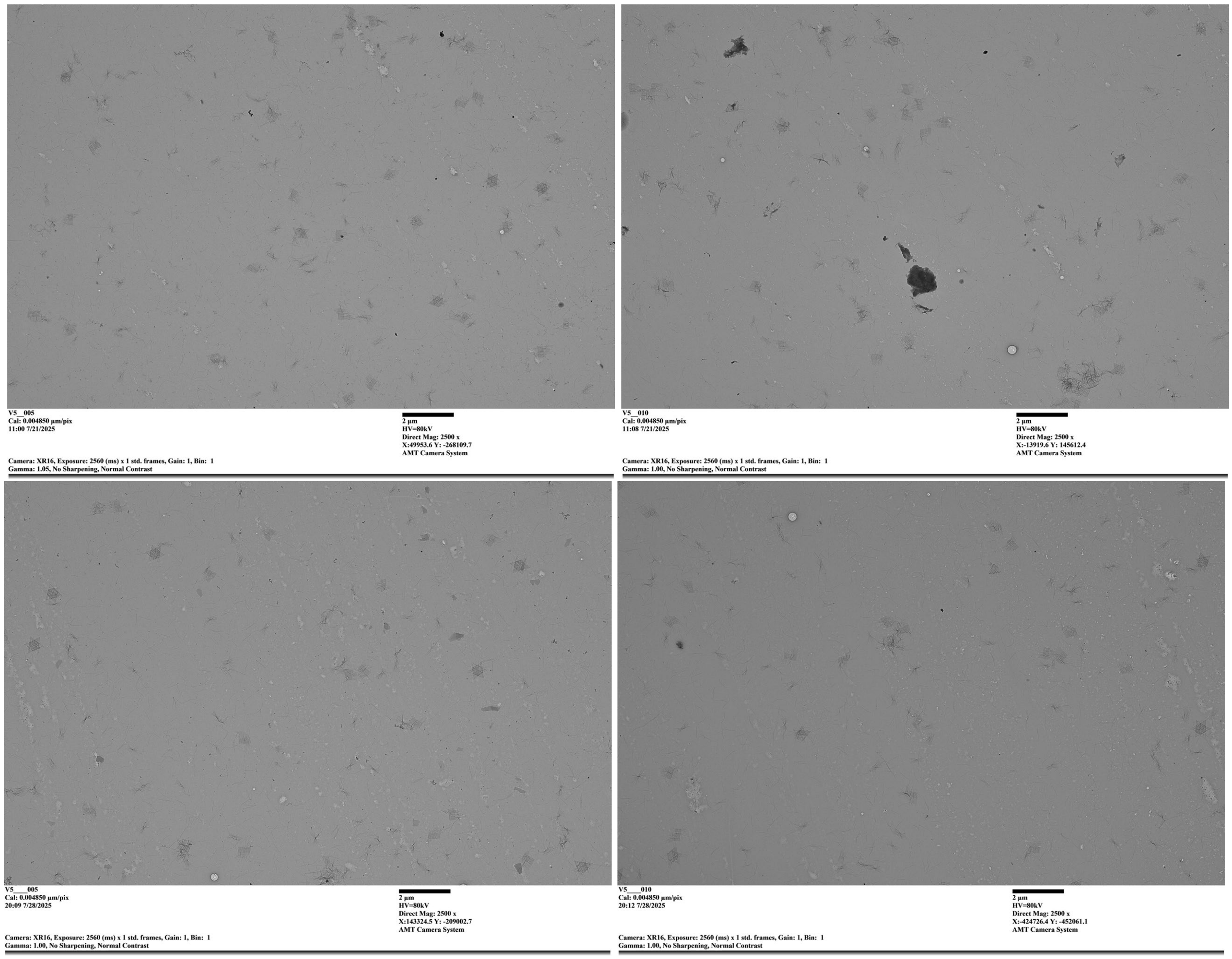

Supp. Fig. 31: Representative TEM images used to quantify the yield and structural completeness of the Sunflower megastructure design with **Loss = 4.7** using the **new** handle library. The corresponding yield data are shown in Figure 6 of the main text.

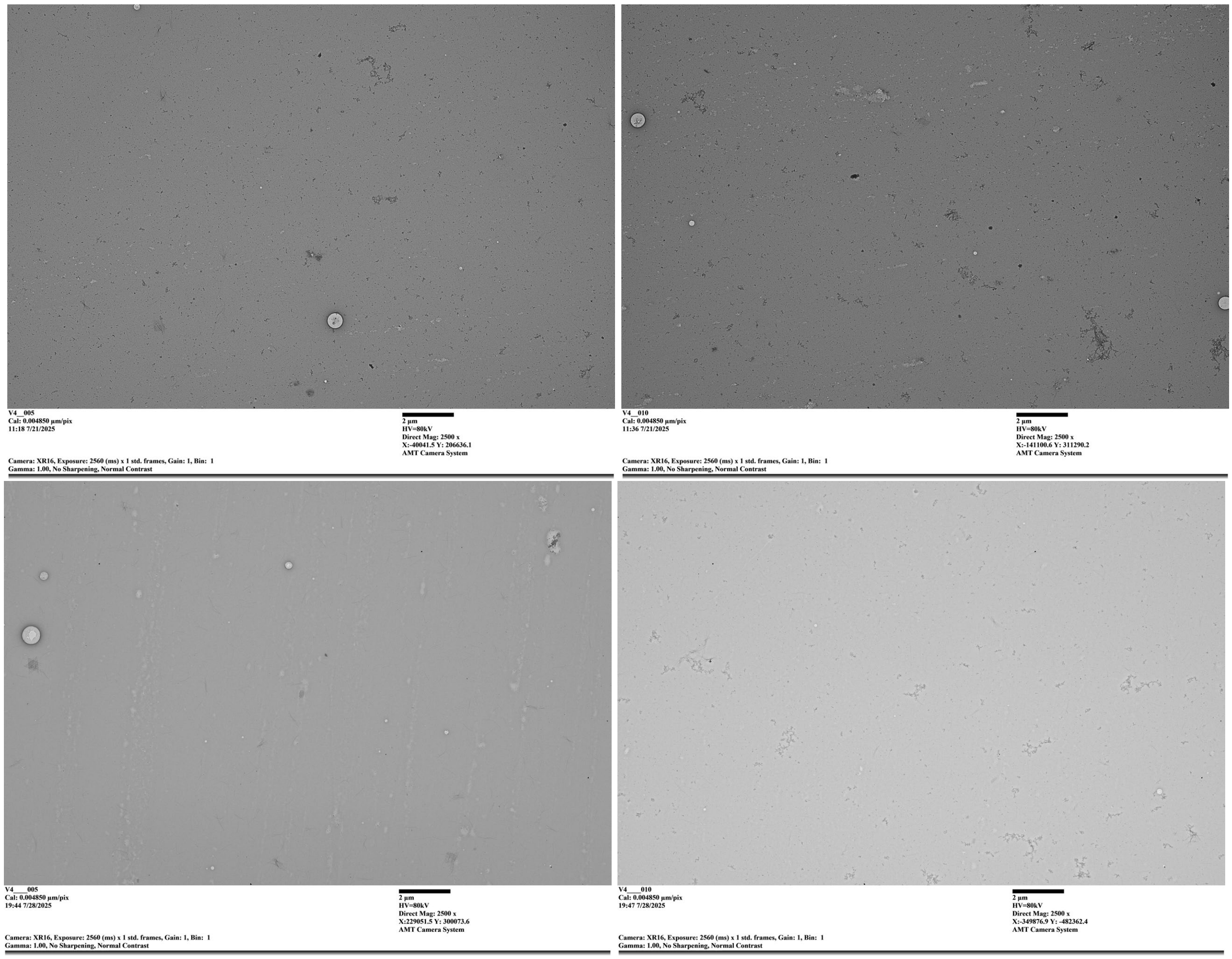

Supp. Fig. 32: Representative TEM images used to quantify the yield and structural completeness of the Sunflower megastructure design with **Loss = 5.1** using the **new** handle library. The corresponding yield data are shown in Figure 6 of the main text.

8.4 Example TEM Images from Figure 7 (ribbon)

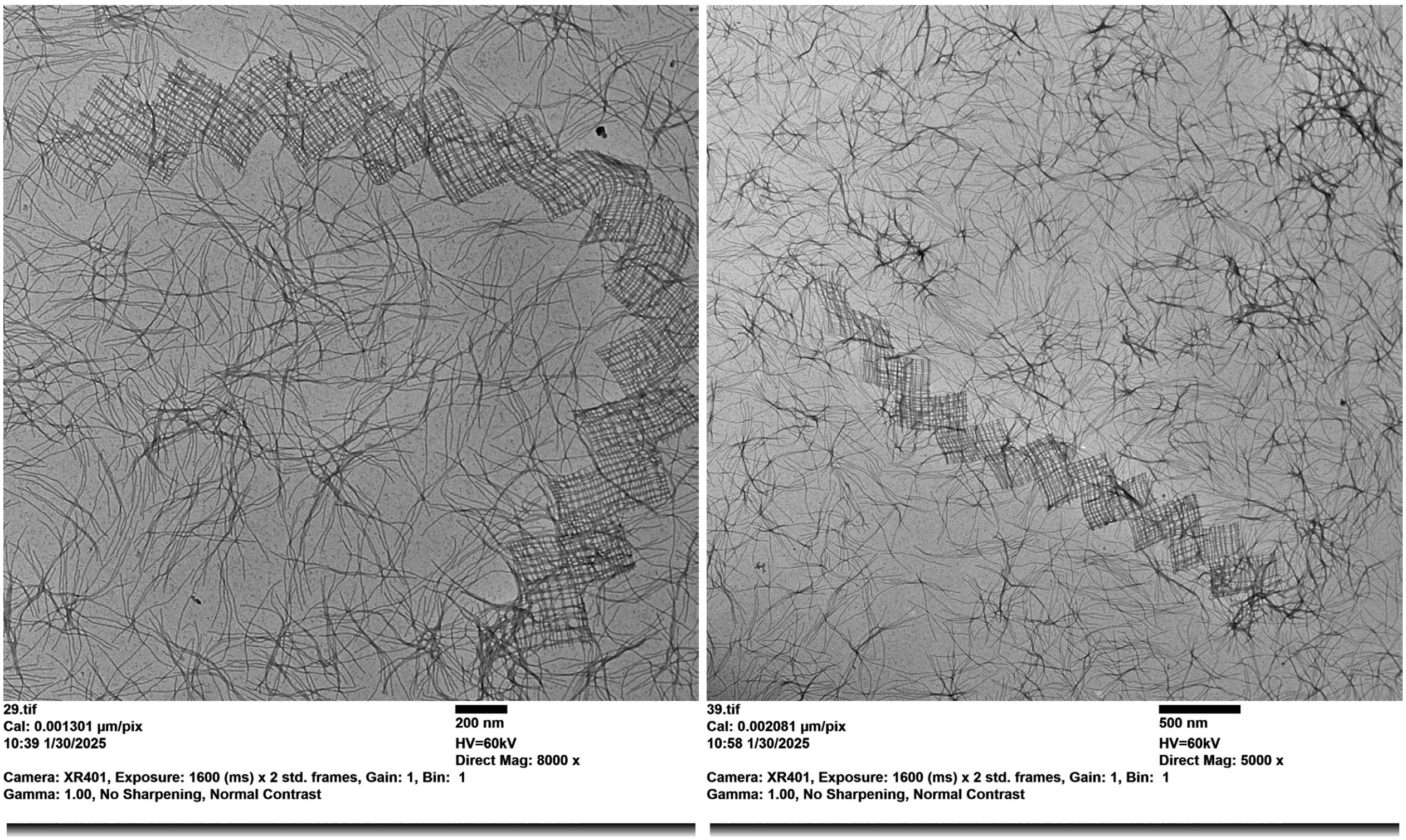

Supp. Fig. 33: Representative TEM images of the infinite ribbon megastructures used to compare the old and new handle libraries. The corresponding count data are shown in Figure 7 of the main text. Both images were taken from the ribbon sample using the new handle library, 15mM $MgCl_2$ and an 8 hour incubation time.
