# Supplementary material for: A computational framework for designing micron-scale crisscross DNA megastructures
Source: Nat Commun. 2026 Jul 17;17:8831. doi: 10.1038/s41467-026-75175-6 (PMC13500642; doi:10.1038/s41467-026-75175-6)
Supplement: Supplementary file 2 [file 41467_2026_75175_MOESM2_ESM.pdf]

Descriptions of Additional Supplementary Files

Supplementary Data 1-

Description:

- The cadnano design and all sequences used for the standard slat origami.
- The cadnano design and all sequences used for the seed origami.
- The plate layouts and sequences for all assembly handles, flat staples and cargo/seed-bearing handles.
- The sequences for all 64 orthogonal handles identified for the new handle library.
- The nanocube design and sequences used.
