# Supplementary material for: A computational framework for designing micron-scale crisscross DNA megastructures
Source: Nat Commun. 2026 Jul 17;17:8831. doi: 10.1038/s41467-026-75175-6 (PMC13500642; doi:10.1038/s41467-026-75175-6)
Supplement: Supplementary file 3 [file 41467_2026_75175_MOESM3_ESM.pdf]

nature portfolio

Peer Review File

A computational framework for designing micron-scale crisscross DNA megastructures

Corresponding Author: Professor William Shih

This file contains all reviewer reports in order by version, followed by all author rebuttals in order by version.

Version 0:

Reviewer comments:

Reviewer #1

(Remarks to the Author)
This manuscript presents a comprehensive computational and experimental framework for the design, optimization, and fabrication of micron-scale crisscross DNA origami megastructures. The authors introduce #-CAD, an open-source graphical design platform that integrates (i) an evolutionary algorithm to minimize parasitic interactions and (ii) a graph-based algorithm to expand and optimize the handle sequence library. Together, these tools significantly improve assembly yield and enable the fabrication of complex multilayer structures that were previously impractical.

The work addresses a critical bottleneck in crisscross DNA origami: the combinatorial complexity of handle assignment and the resulting parasitic interactions that limit assembly yield. The combination of computational design, automation-ready workflows, and systematic experimental validation represents a meaningful advance for large-scale DNA nanotechnology.

Overall, this is a strong, well-integrated study that bridges algorithm development, software engineering, and experimental validation. We'd like to provide a few comments/suggestions hoping to help improve this manuscript.

Comments/suggestions.
The first two paragraphs in “Principles of Crisscross Assembly” are unnecessary. These principles have already been explained in detail in previous reports. A brief one- to two-sentence summary of the prior work would be sufficient.

The third paragraph should serve as the starting point for the paper’s main findings. The core idea is that assigning a unique sequence to every location is not feasible in terms of both cost and design. This constraint leads to the reuse of handles and, consequently, undesired (i.e., parasitic) interactions. Because this key point is repeated in the final paragraph, it would be more effective to introduce it earlier in the manuscript to provide a clearer framing of both the problem and the proposed solution.

The authors present two solutions, one of which is sequence library expansion. However, this approach is not substantial enough to warrant a paper on its own. The manuscript should instead focus on the central contribution: the algorithm designed to minimize parasitic interactions and the accompanying open-source software package. Increasing the number of handles is an obvious strategy and is unlikely to motivate end users to adopt the crisscross platform, as it entails higher upfront costs. Furthermore, the evolutionary algorithm used to minimize parasitic interactions may not be optimal for smaller handle sets.

The 3D-printing workflow is an excellent analogy for the crisscross assembly pipeline. Since the authors introduced this analogy, it would benefit readers if a corresponding 3D-printing schematic were included in Figure 2. For example, Figures 2a and 2b could depict a 3D CAD engine such as Autodesk Fusion, and Figure 2c could include G-code snippets to parallel 3D printer instructions.
The CAD software is well designed and intuitive—excellent work.

The authors claim that the evolutionary algorithm for minimizing parasitic interactions provides a significantly more efficient path toward the final megastructure compared to random selection. However, no direct comparison between random selection and algorithmic selection is presented. This is a major oversight. For at least one medium-complexity structure in

Figure 6, the authors should compare a randomly selected handle set with one selected by the evolutionary algorithm and determine conclusively whether the algorithm truly outperforms random selection.

The reviewer also finds the assigned "Loss" function for the parasitic interaction algorithm to be flawed. There are far more partial mismatches than perfect matches, and using only full matches as the guiding principle for handle selection may not yield optimal structures. Instead, the authors should consider using Hamming distance to quantify "Loss." For example, the sequence AGC TACG can bind perfectly to TCG ATGC, but it can also bind with six nucleotides to TCG ATGT. This partial complementarity is not considered during handle selection. Although the yield improvements shown in Figure 5b are promising, they could potentially be enhanced with a more theoretically rigorous loss function. Additionally, the random handle selection baseline is missing from the loss gradient TEM analysis. Where does random selection fall within the "Loss" spectrum? As noted above, if the authors claim that the evolutionary algorithm outperforms random selection, they must demonstrate this explicitly rather than simply screening incoherent loss values from high to low. Furthermore, zipper handles—where 5′ and 3′ ends bind via 90-degree bending—could also be considered when evaluating binding events, even though these interactions are weaker and less well characterized.

While Loss predicts final yield, its relationship to assembly rate remains less clear. Extended Data suggests final yield differences are stable over time, but kinetics may still vary.

The Loss calculation requires screening all slat pairings and alignments. It would be helpful to explicitly discuss computational scaling for designs >500 slats.

The manuscript emphasizes Type-2 elimination, and later shows Type-1 suppression via library design. However, the relative quantitative contribution of Type-1 interactions to yield reduction remains somewhat indirect.

The manuscript could further contextualize how this approach compares quantitatively with other large-scale DNA origami assembly paradigms (e.g., fractal arrays, SSTs).

No reference is made to one of the earliest DNA handle selection algorithms, seqwalk (https://seqwalk.readthedocs.io/en/latest/), in the discussion of the handle selection process. There are notable similarities between the method presented here and seqwalk. The reviewer believes it is necessary to reference this prior work.

Questions for the Authors:
1. Have the authors investigated how the handle distribution design changes when using smaller versus larger sequence libraries per slat handle location?

2. Even after optimization, some parasitic interactions remain. Have the authors implemented an optimized pooling strategy to isolate slats with the highest parasitic interactions and thereby further reduce the overall parasitic interaction matrix?

(Remarks on code availability)

Reviewer #2

(Remarks to the Author)
The authors report a computational framework for the design and fabrication of crisscross DNA megastructures. Handle sequence assignments are optimized by minimizing a thermodynamically derived Loss function, interpreted as an effective parasitic bond count between slats. Assembly yield, quantified by TEM particle counting, is shown to correlate with Loss across four square megastructure designs and a seven-layer Sunflower design. The handle library is expanded from 32 to 64 orthogonal sequence pairs using a graph-based algorithm applied to NUPACK-computed binding energies. The framework is released as open-source software and tested on ten different DNA structures that were experimentally built, including a large Bird design made of 368 slats and measuring approximately 2 µm in size. The topic is relevant to the DNA nanotechnology community, and the pipeline is a useful advance over prior crisscross work. However, several aspects of the methodology and data analysis require clarification before publication.

1. The Loss function derivation assumes all slats of each type are thermodynamically equivalent, and uses a heuristic dimensionless ΔG value of -10. Since slat purification yields are reported to vary between 60-100%, this assumption may not always hold. It would be helpful if the authors discuss under what conditions the Loss metric may fail to predict experimental yield.
2. All yield data are reported as TEM particle counts per image. As deposition efficiency depends on particle size, structurally distinct designs may not accurately reflect solution yield. An absolute yield measure such as gel densitometry should be provided, or this limitation should be discussed more clearly.
3. No statistical tests are reported anywhere in the manuscript. Non-parametric tests with p-values should be added to the yield comparisons in Figures 5 and 6.
4. The bimodal growth stage distribution for the Sunflower is attributed to a kinetic barrier at

the triangulation stage. This interpretation is not directly supported by the presented data. A time-course experiment tracking stage distributions would be needed to support this claim.
5. The convergence criterion in Method 5 is described as running until Loss appeared to plateau. A stopping criterion or the number of generations used per design should be stated for reproducibility.

My recommendation is that it can be accepted with minor revision.

(Remarks on code availability)

Reviewer #3

(Remarks to the Author)
Summary of the Work
This manuscript describes a computational framework designed to overcome a persistent challenge in DNA nanostructure self-assembly: the scalable construction of micron-scale "crisscross" megastructures. The authors address the inherent limitations of non-cooperative interface interactions—which often lead to undesired or off-target assemblies—by building upon cooperative assembly principles. Specifically, this work introduces an algorithmic approach to optimize the distribution of handle-to-handle interactions and utilizes increased short complementary strands to drive the assembly process effectively. The automated design-to-assembly workflow, including the integration with pipetting robots, is a particularly valuable resource that significantly enhances the utility and reproducibility of the method for the broader DNA origami community.

Evaluation of Innovation and Impact
The ability to reliably create large-scale assemblies is a significant hurdle in the field. This paper provides a robust alternative to standard monomer-interface programming.

Significance: The method is highly practical and addresses the difficulty of minimizing non-specific interactions between handles, a known pain point for researchers.

Utility: By integrating the well-established NUPACK tool for ssDNA handle generation into their framework, the authors have created a workflow that is technically sound and highly accessible. I anticipate this method will be widely adopted by the DNA origami and nanostructure community.

Technical Soundness and Reproducibility
Methodology: The algorithmic approach to distributing handle interactions is sound and effectively minimizes the "noise" of non-specific binding that typically plagues large-scale DNA assembly.

Claims and Data: The work provides sufficient evidence to support its conclusions. The analysis and the logic of the underlying algorithms appear to be without flaws.

Reproducibility: Yes, the manuscript provides enough detail regarding the computational framework and the experimental design for the work to be reproduced by other labs.

Conclusion
This is a high-quality study that provides a much-needed tool for the DNA nanotechnology toolkit. It successfully demonstrates a path toward micron-scale structures through a sophisticated understanding of cooperative assembly.

(Remarks on code availability)
Upon review, the code is well-documented and includes a comprehensive README file with clear instructions for installation and execution. I was able to successfully install and run the application, confirming that the code performs as described in the manuscript.

Reviewer #4

(Remarks to the Author)
I co-reviewed this manuscript with one of the reviewers who provided the listed reports. This is part of the Nature Communications initiative to facilitate training in peer review and to provide appropriate recognition for Early Career Researchers who co-review manuscripts.

(Remarks on code availability)

Reviewer #5

(Remarks to the Author)
I co-reviewed this manuscript with one of the reviewers who provided the listed reports. This is part of the Nature Communications initiative to facilitate training in peer review and to provide appropriate recognition for Early Career

Researchers who co-review manuscripts.

(Remarks on code availability)

Version 1:

Reviewer comments:

Reviewer #1

(Remarks to the Author)
The authors have carefully addressed the comments

(Remarks on code availability)

Reviewer #2

(Remarks to the Author)
The authors have adequately addressed all the concerns raised by reviewers. The manuscript can now be accepted for publication in its current form.

(Remarks on code availability)

Reviewer #4

(Remarks to the Author)
I co-reviewed this manuscript with one of the reviewers who provided the listed reports. This is part of the Nature Communications initiative to facilitate training in peer review and to provide appropriate recognition for Early Career Researchers who co-review manuscripts.

(Remarks on code availability)
Based on a careful assessment of the revised manuscript and the accompanying response letter, I recommend acceptance of this paper. The authors have done a commendable job in addressing the feedback, questions, and suggestions raised during the previous round of review, providing detailed, point-by-point responses that thoughtfully engage with each concern. The revisions have meaningfully improved the clarity, rigor, and overall contribution of the work, and I believe the manuscript now meets the standards required for publication. I thank the authors for their diligence and responsiveness throughout the revision process.

Reviewer #5

(Remarks to the Author)
I co-reviewed this manuscript with one of the reviewers who provided the listed reports. This is part of the Nature Communications initiative to facilitate training in peer review and to provide appropriate recognition for Early Career Researchers who co-review manuscripts.

(Remarks on code availability)

Open Access This Peer Review File is licensed under a Creative Commons Attribution 4.0 International License, which permits use, sharing, adaptation, distribution and reproduction in any medium or format, as long as you give appropriate credit to the original author(s) and the source, provide a link to the Creative Commons license, and indicate if changes were made.
In cases where reviewers are anonymous, credit should be given to 'Anonymous Referee' and the source.
The images or other third party material in this Peer Review File are included in the article's Creative Commons license, unless indicated otherwise in a credit line to the material. If material is not included in the article's Creative Commons license and your intended use is not permitted by statutory regulation or exceeds the permitted use, you will need to obtain permission directly from the copyright holder.
To view a copy of this license, visit https://creativecommons.org/licenses/by/4.0/

Detailed point-by-point response to reviewer comments (reviewers #4 and #5 not included as they co-reviewed with other reviewers)

REVIEWER 1	
**Comment made by Reviewer**	**Response**
This manuscript presents a comprehensive computational and experimental framework for the design, optimization, and fabrication of micron-scale crisscross DNA origami megastructures. The authors introduce #-CAD, an open-source graphical design platform that integrates (i) an evolutionary algorithm to minimize parasitic interactions and (ii) a graph-based algorithm to expand and optimize the handle sequence library. Together, these tools significantly improve assembly yield and enable the fabrication of complex multilayer structures that were previously impractical.<br><br>The work addresses a critical bottleneck in crisscross DNA origami: the combinatorial complexity of handle assignment and the resulting parasitic interactions that limit assembly yield. The combination of computational design, automation-ready	We thank the reviewers for their positive assessment of our work. We address and/or implement the reviewers' suggestions in our responses below.

workflows, and systematic experimental validation represents a meaningful advance for large-scale DNA nanotechnology.<br><br>Overall, this is a strong, well-integrated study that bridges algorithm development, software engineering, and experimental validation. We'd like to provide a few comments/suggestions hoping to help improve this manuscript.	
The first two paragraphs in "Principles of Crisscross Assembly" are unnecessary. These principles have already been explained in detail in previous reports. A brief one- to two-sentence summary of the prior work would be sufficient.<br><br>The third paragraph should serve as the starting point for the paper's main findings. The core idea is that assigning a unique sequence to every location is not feasible in terms of both cost and design. This constraint leads to the reuse of handles and, consequently, undesired (i.e., parasitic) interactions. Because this key point is repeated in the final paragraph, it would be	We agree - we might have gone overboard with our explanation of the basic crisscross concept. To address this, we have removed paragraph 2 entirely and added two short sentences to paragraph 1 to improve flow and ensure the essential concepts we later utilize are properly introduced. The paragraph now reads as follows:<br><br>*"Megastructures consist of 450 nm-long six-helix bundle building blocks, which we refer to as slats, arranged in a crisscross configuration. At every crossing point, complementary handle sequences hybridize to form 7 bp duplexes that collectively hold the megastructure together. Each slat has two binding faces along its long axis, both presenting 32 regularly-spaced positions for placing programmable assembly handle sequences. These sequences dictate which slats bind together in the megastructure, effectively encoding the final shape and assembly pathway. Generally, hierarchical assembly approaches often yield partially-completed structures and require*

more effective to introduce it earlier in the manuscript to provide a clearer framing of both the problem and the proposed solution.	*multi-step protocols [22]. In contrast, the crisscross strategy uses a seed to nucleate cooperative growth, effectively resulting in all-or-nothing assembly (Fig. 1a) [18, 19].”*
The authors present two solutions, one of which is sequence library expansion. However, this approach is not substantial enough to warrant a paper on its own. The manuscript should instead focus on the central contribution: the algorithm designed to minimize parasitic interactions and the accompanying open-source software package. Increasing the number of handles is an obvious strategy and is unlikely to motivate end users to adopt the crisscross platform, as it entails higher upfront costs. Furthermore, the evolutionary algorithm used to minimize parasitic interactions may not be optimal for smaller handle sets.	Indeed - our experiments show that reducing Type-2 interactions has the most impact. To make it more clear that the evolutionary algorithm and associated software is the main contribution in this paper, we have made changes to the language in the abstract, the introduction, the handle library expansion section and the conclusion. The new wording further deprioritizes the handle library expansion. Some examples:<br><br>Abstract:<br>*“Here, we develop a unified framework that standardizes the design and fabrication of crisscross megastructures. We use an evolutionary algorithm to optimize handle assignment and minimize parasitic binding between slats. Together with an expanded handle library, the algorithm enabled the assembly of large, multi-layered megastructures that otherwise would be produced at negligible yields.”*<br><br>Main text associated with Figure 7:<br>*“When compared with the assembly yield improvements enabled by our evolutionary algorithm, the results suggest that parasitic interactions are dominated by Type-2 binding, while the weaker Type-1 bonds have a much lesser effect.”*<br><br>Conclusion:

	"The expanded library further reduces parasitic interactions originating from Type-2 binding. Simultaneously, this allowed us to compare the assembly yields between two handle libraries, and thus evaluate the impact of weakening Type-1 interactions. The results showed that the two libraries had roughly equal yields for two different designs, implying that weaker Type-1 binding in an orthogonal library has limited effect on assembly yield. We conclude that preventing Type-2 binding is much more important than Type-1 to improve assembly yield."<br><br>For your second point on smaller handle sets - we address your point in a response to your later question (which resulted in the inclusion of a new supplementary figure).
The 3D-printing workflow is an excellent analogy for the crisscross assembly pipeline. Since the authors introduced this analogy, it would benefit readers if a corresponding 3D-printing schematic were included in Figure 2. For example, Figures 2a and 2b could depict a 3D CAD engine such as Autodesk Fusion, and Figure 2c could include G-code snippets to parallel 3D printer instructions.<br>The CAD software is well designed and intuitive—excellent work.	We thank the reviewer for their encouraging comments.<br><br>As specified, we have expanded Figure 2 to include a comparison 3D printing workflow. We have also extended the caption to highlight the analogous steps for both workflows. This reads as follows:<br><br>"<br>*(j) Standard 3D printing workflow for comparison. From left to right, a workpiece is designed using CAD software (corresponding to panel a). The design is then translated into a print path and converted into machine-readable printer instructions (corresponding to panels b and c). Finally, the workpiece is fabricated by 3D printing (corresponding to panel d).*

	”
The authors claim that the evolutionary algorithm for minimizing parasitic interactions provides a significantly more efficient path toward the final megastructure compared to random selection. However, no direct comparison between random selection and algorithmic selection is presented. This is a major oversight. For at least one medium-complexity structure in Figure 6, the authors should compare a randomly selected handle set with one selected by the evolutionary algorithm and determine conclusively whether the algorithm truly outperforms random selection.	We believe there are two interpretations to this assessment. We will provide a response and edits to the text to both:<br><br>1) Confirmation that a structure optimized with our Loss results in higher yield than a fully random handle assignment.<br><br>It is important to make a good baseline comparison between an optimized and randomly selected handle set before making any assertions on the effectiveness of the algorithm. We actually did do this comparison in Figure 5 but the original presentation obscured this fact as it was only described in Method 18. We now clearly state how the handle assignments were created in the main text:<br><br>*“These included a design with 32 handle pairs and a fully random handle assignment, a second 32-handle-pair design optimized with our algorithm (explained in the subsequent section), and two 64-handle-pair designs optimized to different final Loss values. (Method 18).”*<br><br>2) Efficiency of the algorithm versus direct random handle assignment.<br><br>In Figure 6c, we highlighted regions on the yield plot in which a random handle assignment would realistically be able to achieve the corresponding Loss. However, we realize this comparison was shallow compared to the rest of our analysis. We have now setup a fair

	comparison in Supp. Fig. 4 where we run the algorithm and random handle assignment for the same amount of iterations (100,000 total handle arrays generated) on the Sunflower design. The graph shows a clear improvement in the Loss when using our algorithm while the random generation remains largely stagnant. We have also added the below in the main text to clarify this:<br><br>"<br>*This is in stark contrast to picking the best handle assignment from a large set of random trials, which does not noticeably improve the Loss even after a large number (100,000) of iterations (Supp. Fig. 4).*<br>"
The reviewer also finds the assigned "Loss" function for the parasitic interaction algorithm to be flawed. There are far more partial mismatches than perfect matches, and using only full matches as the guiding principle for handle selection may not yield optimal structures. Instead, the authors should consider using Hamming distance to quantify "Loss." For example, the sequence AGC TACG can bind perfectly to TCG ATGC, but it can also bind with six nucleotides to TCG ATGT. This partial complementarity is not considered during handle selection. Although the yield improvements shown in	We address the various points in the reviewer's comments below:<br><br>1) Alternative Loss functions.<br><br>We agree, a more systematic analysis of the integration of Type-1 interactions into our Loss would have been a valuable addition to the paper. We now have included this exact analysis in Supplementary Note 2, and have compared losses built from a Hamming distance-like metric, full integration of NUPACK energies as well as a nearest-neighbour handle matching metric.<br><br>The results clearly show that Type-1 bonds seem to be much less important than Type-2 bonds in predicting final megastructure yield.

Figure 5b are promising, they could potentially be enhanced with a more theoretically rigorous loss function. Additionally, the random handle selection baseline is missing from the loss gradient TEM analysis. Where does random selection fall within the "Loss" spectrum? As noted above, if the authors claim that the evolutionary algorithm outperforms random selection, they must demonstrate this explicitly rather than simply screening incoherent loss values from high to low. Furthermore, zipper handles—where 5′ and 3′ ends bind via 90-degree bending—could also be considered when evaluating binding events, even though these interactions are weaker and less well characterized.<br>While Loss predicts final yield, its relationship to assembly rate remains less clear. Extended Data suggests final yield differences are stable over time, but kinetics may still vary.	This aligns with our findings in Figure 7 of the main text where we compared our library with one with less orthogonally binding handles and found that the new library had no noticeable impact on yield. The main text now signposts this analysis as below:<br><br>*"An analysis of alternative Loss functions that also include Type-1 parasitic bonds is provided in Supplementary Note 2."*<br><br>2) Random handle assignment baseline in Figure 5b.<br><br>We are aware that Fig. 5b does not provide context on how each handle assignment was obtained. The way we presented our work was to have Fig. 5 focus on the Loss value itself, and Fig. 6 focus on the algorithm. Given that random assignment does not produce a wide range of Loss values, we had to use the algorithm itself to produce the different designs of Fig. 5. The most relevant point in Fig. 5 is that a lower Loss results in a higher yield. We then highlight the difference between Losses obtained with random assignment and our algorithm in Fig. 6. Nevertheless, we have added more detail in the main text to describe the designs of Fig. 5 (see reply to reviewer above) and we have also updated the figure caption with the below:<br><br>*"The design with Loss 7.3 was obtained by a random assignment of 32 handles, while the remaining designs (4.7: 32 handles, 2.6 & 2.1: 64 handles) were optimized through our evolutionary algorithm (illustrated in Fig. 6)."*

<table>
<tr><td></td><td>3) Zipper handles.

In the crisscross origami regime, zipper-like interactions are geometrically impossible because both plus and minus handles extend from their respective slat with the same orientation (3′ -> 5′). In the proposed zipper configuration, the two sequences would have to bend by 90° and align such that their 5′ ends face each other. Since hybridization requires antiparallel strand orientation, this configuration is not possible.

4) Loss vs kinetics.

Indeed, the relationship between Loss and kinetics remains ambiguous. A longer set of experiments and perhaps a new assay would be required since TEM yield analysis is prohibitively time-consuming for experiments that require multiple timepoints. We believe this to be out of scope for this particular work, but it is definitely an interesting avenue of exploration.</td></tr>
<tr><td>The Loss calculation requires screening all slat pairings and alignments. It would be helpful to explicitly discuss computational scaling for designs >500 slats.</td><td>Thanks for drawing our attention to this detail.

The Loss compute time scales quadratically with slat count ($O(n^2)$, where n=number of slats). For designs with equal minus and plus-handle bearing slats, the compute time using our efficient eqcorr2d library is just 2.65s for 2000 total slats on an Apple Silicon M3 Max Mac. We have as yet not designed a structure incorporating more</td></tr>
</table>

	than ~1000 slats, so we believe the algorithm in its current form should be effective for the foreseeable future.<br><br>We have now added an extended analysis to the Methods as follows:<br><br>"<br>*Loss compute time scales quadratically with total slat count ($O(n^2)$). On an M3 Max Mac running macOS Tahoe 26.4.1, we can use eqcorr2d to compute the Loss for a design with 128 slats in 0.01s, and a design with 2000 slats in 2.65s. Evaluating 100,000 different 2000-slat handle assignments for the evolutionary algorithm would take ~3 days on a single thread, but our multi-threaded implementation significantly cuts down the compute time required.*<br>"
The manuscript emphasizes Type-2 elimination, and later shows Type-1 suppression via library design. However, the relative quantitative contribution of Type-1 interactions to yield reduction remains somewhat indirect.	We agree that the relative quantitative contribution of Type-1 interactions to yield reduction is only addressed indirectly in the current manuscript.<br><br>In our experiments the more orthogonal expanded library alone did not lead to a measurable improvement in assembly yield, despite the obvious advantage of the larger sequence pool. In contrast, clear yield improvements were obtained through evolutionary optimization of the handle assignment, which reduces the Type-2-based Loss. Taken together, these results suggest that Type-1 interactions make a smaller contribution to yield reduction under our experimental conditions, while

	Type-2 interactions appear to be the dominant factor. We now make this interpretation more explicit in the revised manuscript.<br><br>As described earlier, we have now made this analysis more explicit at the end of the "Expanded Handle Library" section:<br><br>*"When compared with the assembly yield improvements enabled by our evolutionary algorithm, the results suggest that parasitic interactions are dominated by Type-2 binding, while the weaker Type-1 bonds have a much lesser effect. Nevertheless, expanding to 64 sequence pairs allows for improved performance by enabling the evolutionary algorithm to minimize the Loss even further."*
The manuscript could further contextualize how this approach compares quantitatively with other large-scale DNA origami assembly paradigms (e.g., fractal arrays, SSTs).	This comprehensive comparison with other large-scale approaches has been conducted in detail in the first crisscross origami paper (Multi-micron crisscross structures grown from DNA-origami slats - https://doi.org/10.1038/s41565-022-01283-1, introduction and Supplementary Text 1). We have now added a reference in the text to this previous discussion:<br>"<br>*Further comparisons between crisscross and other hierarchical approaches have been discussed previously [18, 19].*<br>"
No reference is made to one of the earliest DNA handle selection algorithms, seqwalk (https://seqwalk.readthedocs.io/en/latest/), in the discussion of the handle selection	We thank the reviewer for pointing out this similarity to seqwalk. We have now tested sequences generated with seqwalk for orthogonal binding using NUPACK and found that seqwalk is optimized for a different task. Specifically, it generates sequence barcodes in which

process. There are notable similarities between the method presented here and seqwalk. The reviewer believes it is necessary to reference this prior work.	each substring of length *k* occurs at most once. We found that this constraint is not equivalent to enforcing orthogonal binding between sequences.<br><br>We have now included a detailed analysis in Supplementary Note 4 and have added the below note in the main text:<br><br>"<br>*We tested a DNA barcode library generated using the recently developed seqwalk [25] algorithm, but found that barcode orthogonality does not translate into thermodynamic binding orthogonality (Supplementary Note 4). We therefore developed a graph-theory-based algorithm to identify an expanded set of 64 orthogonal sequence pairs.*<br>"
Q: Have the authors investigated how the handle distribution design changes when using smaller versus larger sequence libraries per slat handle location?	This is an interesting question.<br><br>We have now explored this question in greater detail in Supplementary Note 3/Supp. Fig. 2. Specifically, we analyzed the distributions of parasitic bond counts for both random and optimized handle assignments for the Square and the Sunflower. We repeated this analysis for different sequence library sizes.

	We found that larger sequence libraries generally lead to tighter distributions. For the Square with random handle assignments, we further noted that the distribution can be predicted using a combinatorial approach (Supplementary Note 3.1).<br><br>We have also signposted this analysis in the main text as follows:<br><br>"<br>*We continue to observe the same trends when minimizing the Loss for designs with lower handle library sizes (Supp. Fig. 2).*<br>"
Q: Even after optimization, some parasitic interactions remain. Have the authors implemented an optimized pooling strategy to isolate slats with the highest parasitic interactions and thereby further reduce the overall parasitic interaction matrix?	While such a strategy could in principle further reduce residual parasitic interactions, it was not implemented in the present work. In our system, each slat is required for correct megastructure assembly, so simply isolating or withholding individual slats is not straightforward, as this can hinder or prevent completion of the target structure. More generally, one can envision stepwise assembly strategies in which the megastructure is assembled in successive stages, thereby reducing the number of possible parasitic interactions present at each step. Such staging could in principle be designed to separate particularly problematic interactions. We are currently exploring such stepwise assembly protocols, but this lies beyond the scope of the present paper.

REVIEWER 2	
**Comment made by Reviewer**	**Response**
The authors report a computational framework for the design and fabrication of crisscross DNA megastructures. Handle sequence assignments are optimized by minimizing a thermodynamically derived Loss function, interpreted as an effective parasitic bond count between slats. Assembly yield, quantified by TEM particle counting, is shown to correlate with Loss across four square megastructure designs and a seven-layer Sunflower design. The handle library is expanded from 32 to 64 orthogonal sequence pairs using a graph-based algorithm applied to NUPACK-computed binding energies. The framework is released as open-source software and tested on ten different DNA structures that were experimentally built, including a large Bird design made of 368 slats and measuring approximately 2 µm in size. The topic is relevant to the DNA nanotechnology community, and the pipeline is a useful advance over prior crisscross work. However, several aspects of the	We thank the reviewers for their positive assessment of our work. We address and/or implement the reviewers' suggestions in our responses below.

methodology and data analysis require clarification before publication.	
1. The Loss function derivation assumes all slats of each type are thermodynamically equivalent, and uses a heuristic dimensionless ΔG value of -10. Since slat purification yields are reported to vary between 60-100%, this assumption may not always hold. It would be helpful if the authors discuss under what conditions the Loss metric may fail to predict experimental yield.	Your assessment of PEG purification yield is correct. We in fact counter this by measuring the purified slats via Nanodrop and adjusting their volumes in the final assembly mixture accordingly.<br><br>We have now added a clarifying sentence to the Methods:<br>"<br>*Since we typically find variations in slat yields ranging from 60-100%, we use the Nanodrop-measured slat concentrations as the starting point for the megastructure assembly protocol in order to obtain equimolar individual slat concentrations in the final assembly mix.*<br>"<br><br>Your second comment on when our Loss fails to predict experimental yield is also very interesting - we have now added an extended analysis in Supplementary Note 1.6: 'Predictive Limitations'. We add a note on this in the main text:<br><br>*"A derivation of the Loss, together with a more detailed discussion of its interpretation as an effective parasitic interaction bond count is provided in Supplementary Note 1. This metric is best viewed as an intuitive aid rather than a quantity with a direct physical analogue."*

2. All yield data are reported as TEM particle counts per image. As deposition efficiency depends on particle size, structurally distinct designs may not accurately reflect solution yield. An absolute yield measure such as gel densitometry should be provided, or this limitation should be discussed more clearly.	We agree with the reviewer that TEM particle counts per image do not provide an absolute solution-phase yield and can be influenced by deposition behavior on the TEM grid. In particular, structurally distinct particles may differ in diffusion and sedimentation, which could affect grid deposition efficiency. We therefore interpret these measurements as a comparative imaging-based yield metric rather than a direct concentration measurement in solution.<br><br>For the micron-scale DNA megastructures studied here, gel electrophoresis is not a practical alternative because the assembled structures are too large to migrate reliably through standard gel matrices. As a result, particle counting in TEM images was the only feasible standard quantification method available for comparing these designs. Further studies could consider developing a bespoke assay that can directly quantify megastructure yield via fluorescence or some other readout.<br><br>We have now clarified this limitation explicitly in the Methods section:<br><br>"<br>*We note that the particle counts obtained from TEM images should be interpreted as a relative comparison between samples rather than as an absolute measurement of solution-phase yield. The number of particles observed per image depends not only on the concentration of assembled megastructures in solution, but also on their deposition efficiency on the TEM grid. This deposition efficiency may vary with particle size, geometry, and compactness, since such properties can*

	influence diffusion and sedimentation during sample preparation. Consequently, structurally distinct designs may not be directly comparable in terms of absolute concentration based on TEM counts alone. However, because all four Square samples were prepared, purified, deposited, stained, and imaged under the same conditions, we consider the resulting particle counts to provide a useful comparative measure of assembly performance within this experiment. Alternative approaches such as gel densitometry were not feasible here, as the micron-scale DNA megastructures are too large to migrate reliably through standard gel pores.<br>*"*
3. No statistical tests are reported anywhere in the manuscript. Non-parametric tests with p-values should be added to the yield comparisons in Figures 5 and 6.	We thank the reviewer for this suggestion. We performed Spearman's rank correlation analysis for the loss-yield data and now provide concrete statistical evidence that Loss is strongly correlated with the measured yield. The results of these statistical tests are now reported in the figure captions:<br><br>Fig. 5: "*The Loss was strongly negatively correlated with the measured number of structures per image across all images (Spearman's rank correlation, $n = 212$ images, $\rho = -0.9141$, $p = 2.707 \times 10-84$).*<br>*"*<br><br>Fig. 6: "T*he Loss was strongly negatively correlated with the number of fully formed structures per image (Spearman's rank correlation, $n = 150$ images, $\rho = -0.9677$, $p = 1.984 \times 10-90$).*<br>*"*

4. The bimodal growth stage distribution for the Sunflower is attributed to a kinetic barrier at<br>the triangulation stage. This interpretation is not directly supported by the presented data. A time-course experiment tracking stage distributions would be needed to support this claim.	We thank the reviewer for this important point. We agree that the presented data do not directly demonstrate that the bimodal growth-stage distribution arises from a kinetic barrier specifically at the triangulation stage. Establishing this mechanism conclusively would indeed require a time-course experiment that tracks the stage distribution during assembly.<br><br>We have therefore revised the text to soften this interpretation. The manuscript now states the observed bimodal distribution as an experimental result and presents the connection to triangulation only as a possible explanation rather than a demonstrated mechanism:<br><br>*"Interestingly, for each design, most structures were found either in growth stage 1 or fully completed, resulting in a bimodal distribution. This pattern suggests that assembly may encounter a kinetic bottleneck after stage 1. We speculate that this bottleneck could coincide with the onset of triangulation during growth i.e. the point at which the structure must rigidify into a triangular grid to allow additional slats to bind and continue assembly. However, the present data do not directly establish this mechanism."*
5. The convergence criterion in Method 5 is described as running until Loss appeared to plateau. A stopping criterion or the number of generations used per design should be stated for reproducibility.	We agree that the stopping criterion should be described more explicitly for reproducibility.<br><br>For each design shown, we have provided the Loss value obtained in either the main or supplementary text, as well as the optimized handle

	arrays. Given that the Loss typically converges to a plateau rather than a single optimal assignment, the exact stopping point will have minimal impact on the final Loss, and different random seeds will also induce minor variations. As long as users employ one of the optimized parameter sets described in Extended Data Figs. 2 & 3, they are guaranteed to follow the same evolution trajectory and obtain similar results to the ones we obtained for Fig. 4.<br><br>Furthermore, it was difficult to pin down an exact convergence criterion that was valid for all designs considered, as some could be optimized within a few hundred generations while others required 1000-2000 generations to reach a plateau point. For consistency, we evolved all designs for at least 1000 generations and ensured that at least the two highest bond counts were eliminated from an initial random handle assignment. We did not track the exact number of generations and instead recorded the final Loss and linked handle assignment for each design.<br><br>To clarify the above, Method 5 now reads as follows:<br><br>"<br>*For each design presented in Fig. 4, we used the evolutionary algorithm to optimize the handle assignments. All evolution trajectories roughly followed those illustrated in Fig. 6b before reaching a plateau point, with some variation in how long it took to completely eliminate the current highest bond count. Rather than apply an explicit stopping criterion, we ran the algorithm until it appeared to plateau, typically for*

	more than 1000 generations, and at least a 2–3 unit improvement in the Loss compared to the initial random assignment was obtained. The parameters followed the best practices shown in Extended Data Fig. 2, although minor variations were used between designs to reduce runtimes.<br>*"*
**REVIEWER 3**	
**Comment made by Reviewer**	**Response**
This manuscript describes a computational framework designed to overcome a persistent challenge in DNA nanostructure self-assembly: the scalable construction of micron-scale "crisscross" megastructures. The authors address the inherent limitations of non-cooperative interface interactions—which often lead to undesired or off-target assemblies—by building upon cooperative assembly principles. Specifically, this work introduces an algorithmic approach to optimize the distribution of handle-to-handle interactions and utilizes increased short complementary strands to drive the assembly process effectively. The automated design-to-assembly workflow, including the integration with pipetting robots, is a particularly valuable resource	We thank the reviewers for their positive assessment of our work!

that significantly enhances the utility and reproducibility of the method for the broader DNA origami community.<br><br>The ability to reliably create large-scale assemblies is a significant hurdle in the field. This paper provides a robust alternative to standard monomer-interface programming.<br><br>Significance: The method is highly practical and addresses the difficulty of minimizing non-specific interactions between handles, a known pain point for researchers.<br><br>Utility: By integrating the well-established NUPACK tool for ssDNA handle generation into their framework, the authors have created a workflow that is technically sound and highly accessible. I anticipate this method will be widely adopted by the DNA origami and nanostructure community.<br><br>Methodology: The algorithmic approach to distributing handle interactions is sound and effectively minimizes the "noise" of non-specific binding that typically plagues large-scale DNA assembly.	

Claims and Data: The work provides sufficient evidence to support its conclusions. The analysis and the logic of the underlying algorithms appear to be without flaws.<br><br>Reproducibility: Yes, the manuscript provides enough detail regarding the computational framework and the experimental design for the work to be reproduced by other labs.<br><br>This is a high-quality study that provides a much-needed tool for the DNA nanotechnology toolkit. It successfully demonstrates a path toward micron-scale structures through a sophisticated understanding of cooperative assembly.	
